# Simultaneous Effects of Single-Nucleotide Polymorphisms on the Estimated Breeding Value of Milk, Fat, and Protein Yield of Holstein Friesian Cows in Hungary

**DOI:** 10.3390/ani14233518

**Published:** 2024-12-05

**Authors:** László Bognár, Zsolt Jenő Kőrösi, Szabolcs Albin Bene, Ferenc Szabó, István Anton, Attila Zsolnai

**Affiliations:** 1 Association of Hungarian Holstein Breeders, Lőportár utca 16., H-1134 Budapest, Hungary; bognar@holstein.hu (L.B.); korosi@holstein.hu (Z.J.K.); 2 Albert Kázmér Faculty of Mosonmagyaróvár, Széchenyi István University, Vár tér 2., H-9200 Mosonmagyaróvár, Hungary; szabo.ferenc@sze.hu; 3 Institute of Animal Husbandry Sciences, Hungarian University of Agriculture and Life Sciences, Guba Sándor utca 40., H-7400 Kaposvár, Hungary; bene.szabolcs.albin@uni-mate.hu (S.A.B.); anton.istvan@uni-mate.hu (I.A.)

**Keywords:** Holstein, genome-wide association, single-nucleotide polymorphism, estimated breeding value

## Abstract

This study aimed to find SNPs that have an effect on the estimated breeding values (EBVs) of milk (MY), fat (FY), and protein yield (PY) of Holstein Friesian cows in Hungary. Holstein Friesian cows (n = 2963) were genotyped on a Eurogenomics (EuroG_MDv4) chip. The EBVs for MY, FY, and PY were obtained from the Association of Hungarian Holstein Breeders (AHHB). The loci associated with the EBVs were identified via three approaches: the calculation of genetic distance of the SNPs (*F_st_marker_*), linear regression, and haplotype association tests. Nine SNPs were significantly associated with MY, FY, and PY located on BTA 2, 5, 28, and X. Among the nine SNPs identified, BTB-00219372 on BTA 5 had a positive β coefficient for MY and a negative β coefficient for FY and PY. In addition, BovineHD3000027615 on BTA X had a positive β coefficient for both MY and PY, as well as a negative β coefficient for FY. The identified SNPs were located near several genes that remain unstudied in cattle, which are potential targets for closer scrutiny in relation to milk properties. The markers associated with two or three EBVs could be used in selection with high efficiency to accelerate genetic development and help AHHB experts achieve their breeding. Most marker effects point in the same direction on EBVs; however, we found that BTB-00219372 and BovineHD3000027615 could be used with caution to increase one EBV while decreasing the other EBV or EBVs.

## Simple Summary

By analyzing the genome of Hungarian Holstein Friesian cows, we looked for genomic regions which have an effect on the milk, fat, and protein yield. Among the sampled animals and the investigated nucleotides, nine were simultaneously associated with milk, fat, and protein yield. Among the nine variants, two had opposite effects; for example, while increasing the value of milk yield, the other one or two parameters’ values decreased. The acquired knowledge can help in the planning of breeding schemes to avoid unwanted interactions among the abovementioned yield parameters.

## 1. Introduction

The Holstein Friesian (HF) is perhaps the most recognized and widely distributed dairy cattle breed worldwide, originating in the Netherlands, where black Batavian and white Friesian animals were crossed to create a new breed with superior milk-producing abilities, even under limited feed resources [1]. The Holstein breed rapidly spread to North America in the 1800s and later to the entire world [1].

In 1960, The World Holstein Friesian Federation was founded to improve, develop, and promote the HF breed, with the first international Holstein meeting held in 1964 [2]. The HF breed was introduced to Hungary in the 1970s, and the Association of Hungarian Holstein Breeders (AHHB) was founded in 1989. At present, the AHHB has more than 900 members possessing over 248,000 cows [3].

Advances in molecular genetics, especially in the typing of single-nucleotide polymorphisms (SNPs) using the microarray technique, and the availability of genomic data on many individuals have radically changed the entire dairy sector worldwide [4]. DNA microarray-based genomic investigations on Holsteins in the United States started in 2009 and were quickly recognized as a valuable tool for selective breeding [5].

In the last few years, several genome-wide association studies (GWASs) were performed to identify the loci associated with different production traits in HF cattle. For instance, in Chinese HF, several SNPs were linked to various milk production traits [6], milk fatty acid [7], milk protein composition and/or protein percentage [8], udder health, and conformation [9,10]. In Irish Holsteins, novel SNPs have been associated with milk production using advanced statistical methods [11]. In Nordic Holsteins, SNPs were revealed [12] affecting both milk production and mastitis resistance. A large-scale GWAS in US Holsteins has confirmed previously identified SNPs and uncovered new genetic effects on various milk production traits [13]. Another large-scale study in European countries identified regions on the Bos taurus genome associated with milk yield (MY) and lactation curve parameters, supporting both known and novel candidate genes for MY in HF cows [14].

In Hungary, Anton et al. [15] investigated the effect of the lysine/alanine (K232A) polymorphism at the *DGAT1* locus on milk production traits of HF cows. The first GWASs related to cattle production in Hungary were published in 2018, identifying several loci associated with the breeding values for fertility and beef [16]. A major breakthrough in Hungarian HF came with the introduction of genomically enhanced breeding value (GEBV) estimation in 2017, which blended the genomic best linear unbiased prediction with traditional breeding values [17], followed by the HUNGENOM project in 2019, providing a genomic selection tool for Hungarian breeders based on genomic breeding value estimation [3].

This study, based on the data collected in the HUNGENOM project, aimed to reveal the combined effects of SNPs on the previously calculated EBVs for milk (MY), fat (FY), and protein yield (PY) in Hungarian HF cows.

## 2. Materials and Methods

This study did not require approval from the Ethical Committee on Animal Experiments, since genotyping is part of the routine breeding procedure of the AHHB. All data concerning the phenotype and genotype of animals were provided by the AHHB. HF cows were genotyped using the EuroG_MDv4 microarray chip (Eurogenomics, Amsterdam, The Netherlands) containing 67,227 SNPs. After including only samples with a call rate greater than 0.95 and SNPs with a call rate above 0.95, as well as removing duplicated samples, the final dataset comprised 2963 cows and 59,151 SNPs.

EBVs were calculated by the AHHB using 40,947 SNPs based on the genomic breeding value estimation method specified for Hungarian data [18], excluding SNPs located on chromosome X. The genomic prediction model was based on the Bayesian multi-QTL model [19], where the effects of dense SNPs across the whole genome were fitted directly without using haplotypes or identical-by-descent probabilities [20]. Although the method can be applied for multiple traits simultaneously, the routine genomic evaluations are single-trait analyses, i.e., *m* = 1. For m traits, the following model was applied:

(1) $$y_{i}=\mu +u_{i}+\sum_{j=1}^{40947}\;z_{\mathrm{ij}}\;q_{j}\;v_{j}+e_{i}$$

where *y*_i_ is the (*m* × 1) vector of phenotypes (deregressed proofs), *u*_i_ is the (*m* × 1) vector of random polygenic effects, and *e*_i_ is the (*m* × 1) vector of residuals of animal *i*; *μ* is the (*m* × 1) vector of fixed trait means; *q*_j_ is the (3 × 1) vector of random non-scaled SNP effects for SNP *j* ([40947]) with alleles 0 (missing), 1 (A), and 2 (B); *v*_j_ is the (1 × *m*) random scaling vector for SNP *j*; and *z*_ij_ is the (1 × 3) design vector for animal *i* and SNP *j* ([ 0 2 0 ], [ 0 1 1 ], [ 0 0 2 ], or [ 2 0 0 ] for homozygous [AA], heterozygous [AB], homozygous [BB], or non-genotyped [00] animals at SNP *j*, respectively).

**Figure 1:**
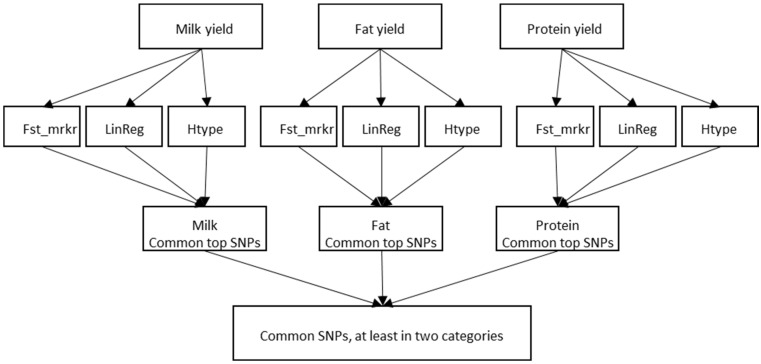
Flow diagram of the search for single-nucleotide polymorphisms (SNPs) associated with two or three of the estimated breeding values (EBVs). First row: EBV values for MY, FY, and PY obtained from the Association of Hungarian Holstein Breeders; second row: the applied tests for each trait (Fst_mrkr: genetic distance of SNPs, LinReg: linear regression, Htype: haplotype association); third row: the top SNPs found using different tests were identified for each trait; fourth row: the top SNPs shared by two or three traits were identified.

For the GWASs, the animals were divided according to their EBVs for MY, FY, and PY (EBV_milk_, EBV_fat_, EBV_prot_, respectively). First, in each EBV category, high and low valued groups were created as follows: EBV_milk_high_ > 1465, EBV_milk_low_ < 328; EBV_fat_high_ > 65, EBV_fat_low_ < 19; EBV_prot_high_ > 51, EBV_prot_low_ < 21. For each trait (EBV_milk_, EBV_fat_, EBV_prot_), three independent approaches (genetic distance of the SNPs (Fst_marker), linear regression [21], and haplotype association tests [22]) were calculated using the SNP and Variation Suite 8.8.1 (SVS) software (Golden Helix, Bozman, MT, USA). The flow diagram of the procedure is illustrated in Figure 1.

**Figure 2:**
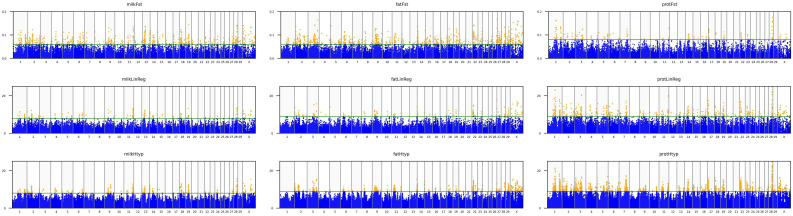
Manhattan plots of the associations of EBVs for milk (EBV_milk_; **left**), fat (EBV_fat_; **middle**), and protein (ENV_prot_; **right**) with *F*_st_marker_ (**top row**), linear regression (**middle row**), and five-SNP haplotypes (**bottom row**). The green lines are the thresholds above which the markers are considered as top hits.

In haplotype association tests, the window size was set to 5 markers; a chi-squared test was performed for each haplotype. The haplotypes were constructed using the expectation maximization (EM) algorithm (maximum EM iteration = 50, EM convergence tolerance = 0.0001). After visual inspection of the Manhattan plots (Figure 2) of the EBV_milk_, EBV_fat_, and EBV_prot_ associations, the threshold values for *F_st_marker_* and –log_10_(*p*) in linear regression or haplotype association were 0.06, 8, and 8 for EBV_milk_; 0.06, 9, and 9 for EBV_fat_; and 0.08, 9, and 9 for EBV_prot_, respectively. The SNPs above the thresholds were determined for each trait (third row in Figure 1), and those associated with at least two traits were identified (fourth row in Figure 1). The false discovery rates [23] of the identified 74 SNPs ranged from 1.3 × 10^−21^ to 6.0 × 10^−06^. Indices were created for the *F_st_marker_*, linear regression, and haplotype association analysis results by rescaling the values from 0 to 1 and averaging them.

The *B. taurus* genome assembly ARS-UCD1.2 was used to look for genes located ±1 million base pairs (Mbp) from the common hits (Table 1 and Table S1). When no gene was mapped within ±1 Mbp, the distance was extended to ±3 Mbp.

## 3. Results

Five SNPs associated with EBVs for MY and FY were identified on BTAs 9, 18, and 19. In addition, we discovered 44 SNPs associated with EBVs for MY and PY located on BTAs 1–6, 11, 13–15, 18, 19, 24, 28, and X. Moreover, 16 SNPs were related to EBVs for FY and PY on BTAs 3, 11, 19, 22, and X. Furthermore, nine SNPs were associated with EBVs for MY, FY, and PY located on BTAs 2, 5, 28, and X (Table 1, Supplementary Table S1). The maximum values of the identified SNPs were 0.17 for *F*_st_marker_, 24.9 for the –log_10_(*p*) of the linear regression, and 26.4 for the –log_10_(*p*) of the haplotype association. Among our findings, the most prominent hits, mean values >0.8 (Supplementary Table S2), were located on BTAs 2, 11, 19, 28, and X for MY; BTAs 3, 22, 28, and X for FY; and BTAs 1 and 28 for PY. Among the nine SNPs associated with the EBVs for MY, FY, and PY, seven were among the top (means > 0.8), three of them within 1.18 million base pairs on BTA 28.

### 3.1. SNPs Associated with Two EBVs and Their Surrounding Genes

The genes found in the vicinity of the top SNPs examined are summarized in Supplementary Table S1; the descriptions and references of them are given in Supplement File S1.

### 3.2. SNPs Associated with EBV_milk_, EBV_fat_, and EBV_prot_ and Their Surrounding Genes

Nine SNPs on BTAs 2, 5, 28, and X were associated with EBV_milk_, EBV_fat_, and EBV_prot_.

No genes were within ±3 Mbp of the two SNPs on BTA 2 (Supplementary Table S1). The one SNP on BTA 5 was near two genes: *PPFIA2* and *METTL25*. On BTA 28, we identified three genes (*CCSER2*, *SHLD2*, and *ANXA8L1*), while on chromosome X, the abundance of the genes was elevated.

## 4. Discussion

This study aimed to identify the SNPs associated with ENVs for two or three milk traits. It used three algorithms to identify the associated SNPs (Figure 1) and used the top hits (Figure 2) to identify the candidate genes. Many of the surrounding genes have already been studied in *B. taurus*, *B. indicus*, or *Bos grunniens* and are associated with milk characteristics, weight, collagen synthesis, or sperm quality.

On BTA 5, *PPFIA2* has been associated with regulation of the reproduction process [24]. The gene was identified as a candidate gene for 305-day MY in Guzerá cattle [25]. Furthermore, it was also associated with MY and FY in several Thai dairy cattle populations [26]. *METTL25* was identified as a candidate gene for claw disorder digital dermatitis in HF and Simmental cows and may affect disease resistance [27].

On BTA 28, mutations in *CCSER2* were found to affect the fat, protein, casein, and lactose traits of Gannan yak milk [28]. SHLD2 is an effector of transformation-related protein 53 binding protein 1 (TRP53BP1/53BP1) and was critical in suppressing large deletions within the immunoglobulin heavy-chain locus in mice [29]. The gene ontology annotations associated with ANXA8L1 included calcium ion binding and calcium-dependent phospholipid binding [30].

On BTA X, *FMR1* has been associated with bull fertility traits [31] and fragile X syndrome [32]. FMR1NB has an unknown function that is predicted to be a membrane protein [33]. *AFF2* is implicated in fragile X syndrome in Nelore cattle [34]. Mutations in *IDS* cause mucopolysaccharidosis type II, also known as the Hunter syndrome [35]. *AKAP4* is expressed in various tissues and may play a role in defects in sperm flagellum and motility [36]. *CCNB3* is expressed in various tissues and is indispensable for female fertility in mice [37]. *DGKK* was associated with hypospadias in humans and Holstein cattle, which is a congenital defect of the genital region [38]. SNPs in *SHROOM4* have been associated with intellectual developmental disorder and epilepsy [39]. Mutations in *BMP15* have been associated with fresh sperm motility in Holstein bulls, making it a potential marker for sperm quality [40]. *NUDT10* and *NUDT11* are luteinizing hormone-regulated genes in bovine granulosa and have major roles in ovarian function in Holstein cows [41]. *CXHXorf67* is associated with endometrial stromal sarcomas [42]. GSPT2 is involved in translation termination and mRNA decay. It may be involved in mRNA stability [43]. *MAGED1* and *MAGED4B* showed very high expression during estrus [44]. Mutations in *HEPH* can cause severe microcytic anemia in mice [45]. VSIG4 was associated with macrophage activation by regulating the pyruvate metabolism of mitochondria [46]. *MSN* has roles in lymphocyte homeostasis and primary immunodeficiency diseases [47]. LAS1L may be involved in neurogenetic disorders in humans [48]. ZC3H12B is involved in the proinflammatory activation of macrophages [49]. *ZC4H2* has been identified as a candidate gene for semen quality and fertility in Egyptian buffalo bulls [50]. ASB12 may play a role in muscle fiber growth in different cattle breeds [51]. AMER1 is a potential candidate gene for X-linked hereditary diseases in cattle [52]. *ARHGEF9* is a potential candidate gene for X-linked hereditary diseases and cognitive impairment in cattle [52]. In humans, SPIN4 is associated with overgrowth syndrome and hyperekplexia [53]. Markers covering the ZXDB region were highly differentiated in German Mutton compared to Dorper and Sunit sheep [54]. *ZNF674* is implicated in non-syndromic X-linked cognitive disabilities in humans [55].

In our study, *DGAT1* was not among the top hits due to our filtering setup of the association results. As shown in Figure 2, the applied threshold on the Manhattan plots for PY did not allow the peak at the beginning of BTA 14 to be included in our comparison. Regarding FY, of the algorithms and filters applied, only haplotype regression identified the *DGAT1* region. However, this region was retained among the top hits for MY. On BTA 2, two SNPs associated with the EBVs for MY, FY, and PY (Hapmap47966-BTA-47563, ARS-BFGL-NGS-113042) were 2 Mbp from markers associated with MY reported by Minozzi [56].

Atashi et al. [14] identified three regions on Bos taurus autosome (BTA) 14 containing SNPs associated with MY. Our combined hits associated with EBVs for at least two of the examined traits were not within 3 Mbp of these genes. Jiang et al. [6] identified several SNPs associated with multiple milk production traits in Chinese Holsteins, where three SNPs on BTA14 were associated with MY, FY, and PY. Meredith et al. [11] identified no significant SNPs associated with MY, FY, and PY in Irish HF cows. In the Hungarian Holstein population, we did not find SNPs on BTA14 associated with these traits simultaneously.

Jiang et al. [13] reported three genes (solute carrier family 4 member 4 [SLC4A4], ADAM metallopeptidase with thrombospondin type 1 motif 3 [ADAMTS3], and GC vitamin D binding protein [GC]) on BTA6 that had significant additive effects on the MY and PY of US Holstein cattle. In addition, two SNPs on BTA5 (rs41257416 [position: 105,870,613] and rs110000229 [position: 105,804,923]), located very close to our SNPs, had significant additive effects on PY. Pedrosa et al. [57] described 98 genes located on BTA14 associated with milk production traits (MY, FY, PY, FP, and PP) in North American Holstein cattle. A review article published by Bekele et al. [58] mentioned 136 SNPs significantly associated with two or more milk production traits in Holstein cattle and crossbreds. Out of them, fifty-three, eighteen, ten, and seven SNPs were located on BTAs 14, 6, 20, and 1, respectively. Our top SNPs associated with three traits were located on BTAs 2, 5, 28, and X.

Kolenda et al. [59] found that the *PAEP* gene (beta-lactoglobulin) was associated with MY, FY, and PY. In our study, we identified a marker close to this gene, which was associated with EBV_fat_ and EBV_prot_. Regarding EBV_milk_, the closest marker on BTA11 was 5.5 Mbp from *PAEP* (Supplementary Table S1).

*PPFIA2* has been identified as a candidate gene in connection with MY and FY [25,26], and it takes part in the regulation of reproduction [24]. As for PPFIA2, we found a connection not just with EBV_fat_ and EBV_prot_ but with EBV_milk_ as well.

Several genes around the top hits either had not been studied in cattle and/or had an unknown function. In other cases, their described function in other mammals suggests influences on EBVs. We believe all genes near the associated SNPs warrant more detailed functional studies. In Supplementary Table S1, we underlined those candidate genes which take part in calcium transport (*EFCAB10*, *SBSPON*, *JPH1*, *EMC8*, *CACNA1F*, *ANXA8L1*), starred genes with collagen-related processes (TAPBPL*, P4HA3*), and cfm-labeled some genes known to have cilia and flagella functions (*SPACA9*^cfm^, *B9D* ^cfm^, *CCDC13*^cfm^) and/or known to be membrane proteins.

We were also interested in whether the regression coefficients of the SNPs reported here were all positive or negative across the three EBVs. While most regression coefficients were found to be in the same direction (i.e., consistently negative or positive for EBV_milk_, EBV_fat_, and EBV_prot_), two SNPs showed opposite signs. BTB-00219372 on BTA 5 had a positive β coefficient for MY but a negative β coefficient for FY and PY. In addition, BovineHD3000027615 on BTA X had a positive β coefficient for MY and PY but a negative β coefficient for FY (Supplementary Table S2). The first case might reflect cases where cows produce more but slim milk, which can be a problem in profit realization, including calf rearing. The second case means higher MY and PY but lower FY, which might affect the enjoyment value of milk [60] and the quality of the cheese products.

**Figure 3:**
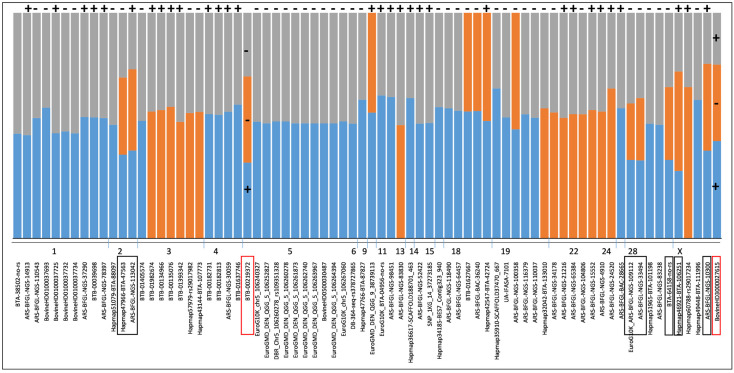
The 74 SNPs associated with EBVs for two or three of the examined traits. The orange (EBV_milk_), blue (EBV_fat_), and gray (EBV_prot_) bars correspond to the regression *β* coefficients of each SNP (Supplementary Table S2). SNPs associated with all three EBVs are shown in boxes. At the top of the figure, the plus and the minus denote all positive or all negative *β* coefficients across traits. Red boxes denote SNPs where the *β* coefficients were opposite, e.g., FY and PY were negative while MY was positive on BTA 5, and FY was negative while MY and PY were positive on chromosome X.

Since not just linear regression was used to determine the common sets of SNPs, we standardized the results of the three algorithms by rescaling their values between 0 and 1 and averaging them for each EBV. The means of the rescaled values for the top alleles associated with EBVs for at least two of the studied traits ranged from 0.445 to 0.9260. The strongest signal was for SNP BTA-64158-no-rs on BTA 28 (Supplementary Table S2, Figure 2) with ENVs for all three examined traits. Supplementary Table S2 and Figure 3 show that 58 SNPs were significantly associated with EBV_milk_ (orange bars), while 16 SNPs were significantly associated with EBV_fat_ (blue bars). We identified 5 SNPs associated with the EBVs for MY and FY (BTAs 9, 18, 19), 44 SNPs associated with the EBVs for MY and PY (BTAs 1–6, 11, 13–15, 18, 19, 24, 28, and X), and 16 SNPs associated with the EBVs for FY and PY (BTAs 3, 11, 19, 22, and X).

## 5. Conclusions

Our study exclusively focused on the SNPs and candidate genes associated with EBVs for two or three milk production traits (MY, PY, and FY) in HF cows in Hungary. Notably, nine SNPs were associated with the EBVs for MY, FY, and PY (BTAs 2, 5, 28, and X). Some of the identified markers were located very near to previously reported chromosome regions or genes that were not previously linked to milk parameters in cattle or linked to other properties. Other genes reported to have an effect on and/or that could be linked to milk properties (*SBSPON*, *KLHL8*, *SLC35A2*, *SLC38A5*, *CTH*, *SPACA9*, *PAEP*, *CTNNB1*, *OXTR*, *PIN4*, *PPFIA2*, and *CCSER2*) were also found in our study (for genes *KLHL8*, *SLC35A2*, *SLC38A5*, *CTH*, *CTNNB1*, *OXTR*, and *PIN4*, see Supplementary Table S1 and Supplement File S1). Several genes like *EFCAB10*, *GLOD5*, *NONO*, and *TMEM70* were reported in this study and have not been previously investigated in connection with milk properties in cattle.

Regarding the marker effects, they exhibited a consistent directional influence across EBVs. However, two markers, BTB-00219372 and BovineHD3000027615, showed diver-gent effects. Since these markers could be used to selectively increase one EBV while potentially decreasing others, special consideration when using these markers in selective breeding programs is required.

These findings provide new information that could accelerate genetic progress and may help specialists from the AHHB achieve their breeding and selection goals in the Holstein population in Hungary.

## Figures and Tables

**Table 1:** The 74 SNPs associated with at least two of the examined traits.

SNP no.	EBV_milk_	EBV_fat_	EBV_prot_
5	+	+	
44	+		+
16		+	+
9	+	+	+
Total	58	30	69

## Data Availability

The raw dataset presented in this article is not readily available because the data are part of ongoing studies and are owned by the Association of Hungarian Holstein Breeders. Requests to access the datasets should be directed to the first author.

## Supplementary Materials

The following supporting information can be downloaded at: https://www.mdpi.com/article/10.3390/ani14233518/s1, Table S1: The names of the markers associated with two or three EBV values, their genomic positions (B. taurus genome build ARS-UCD1.2), and genes found around the markers; Table S2: The means of rescaled values of the applied algorithms and regression beta values of linear regressions on EBVs for MY, FY, and PY; Supplement File S1: SNPs Associated with Two EBVs and Their Surrounding Genes.
