# Supplementary material for: Simultaneous Effects of Single-Nucleotide Polymorphisms on the Estimated Breeding Value of Milk, Fat, and Protein Yield of Holstein Friesian Cows in Hungary
Source: Animals (Basel). 2024 Dec 5;14(23):3518. doi: 10.3390/ani14233518 (PMC11640446; doi:10.3390/ani14233518)
Supplement: Supplementary file 1 [file animals-14-03518-s001.zip › Supplement File S1.pdf]

Supplement File S1. SNPs associated with two EBVs and their surrounding genes

EBV_{milk} and EBV_{fat}

Five SNPs associated with EBV_{milk} and EBV_{fat} were on BTA 9, 18 and 19 (Tables 1 and 2). On BTA 9, tubulin epsilon 1 (*TUBE1*) and major facilitator superfamily domain containing 4B (*MFSD4B*) genes were within ± 1 Mbp of these SNPs. *TUBE1* encodes a protein which plays a central role in organization of the microtubules during centriole duplication. Notably, *TUBE1* on BTA9 was identified as a candidate gene for live weight, net meat weight, average daily gain and carcass weight in Chinese Simmental beef cattle [61].

MFSD4B is predicted to be involved in glucose transmembrane transport and sodium ion transport. Since sugar is used to synthesize fatty acids, sugar transporters major facilitator superfamily domain containing 4A (MFSD4A) and MFSD4B could be candidates for C18:0 regulating loci in Brazilian Holsteins [62].

While the three neighboring SNPs on BTA 18 were not located near any gene, nucleotide-binding oligomerization domain containing 2 (*NOD2*) was located within ±3 Mbp of them, which stimulates host immunity. Yamauchi et al. [63] concluded that consumption of bovine lactoferrin increased *NOD2* expression. A GWAS by Moretti et al. [64] identified *NOD2* as a candidate gene for mastitis resistance in Italian HF cattle.

EBV_{milk} and EBV_{prot}

Of the 44 SNPs associated with EBV_{milk} and EBV_{prot}, 10 were located in four regions on BTA 1 that contained the genes chloride voltage-gated channel 2 (*CLCNC2*), MAP6 domain containing 1 (*MAP6D1*), tetratricopeptide repeat domain 14 (*TTC14*), armadillo repeat containing 8 (*ARMC8*), centrosomal protein 70 (*CEP70*), and DNA topoisomerase II binding protein 1 (*TOPBP1*; Tables 1, 2).

CLCN2 encodes a chloride channel protein that is involved in regulating membrane potential and fluid transport. It has been associated with growth rates in cattle [65].

MAP6D1 encodes a microtubule-associated protein involved in neuronal development and function. *MAP6D1* has been associated with MY and fat percentage (FP) in dairy cattle. MAP6D1 contains a stretch of cysteines that can participate in palmitoylation, the covalent attachment of fatty acids [66] allowing different ways of transports between membrane compartments [67]. A study on carnitine palmitoyltransferase 1 indicates that lipid mobilization in dairy cows could play a role in reproductive performance [68].

TTC14 encodes a protein that is involved in ciliary development and function. It has been associated with ciliary dyskinesia, a disorder that affects the respiratory tract and other organs. TTC14 may also play a role in microtubule assembly and stability [69].

CEP70 encodes a protein that is involved in microtubule organization and dynamics at the centrosome. It has been implicated in breast cancer development and progression, as well as cell proliferation and migration [70]. *CEP70* was found to be expressed in the mammary gland of sheep [71] and might adversely affect milk production and resilience traits in sheep [72].

ARMC8 encodes a protein that is involved in cell–cell adhesion and signaling. It interacts with various proteins at the adherens junctions and desmosomes and may regulate epithelial differentiation and morphogenesis. ARMC8 is also a component of the minor spliceosome, which processes a subset of pre-messenger RNA (mRNA) introns. In human, *ARMC8* may be a potential therapeutic target therapies for bladder cancer or colon cancer [73] [74].

TOPBP1 encodes a protein that is involved in the DNA replication, repair, and damage response. It interacts with various proteins at the replication fork and the DNA damage sites, and activates the ATR kinase, which coordinates the cellular response to DNA damage.

TOPBP1 may also be involved in transcriptional regulation and cell cycle control. It was associated with hoof health in cattle [75].

Clusters of 4, 11, 2, 2, and 4 SNPs on BTAs 4, 5, 14, 18, and 19 were located near the genes putative homeodomain transcription factor 2 (*PHTF2*) and EF-hand calcium binding domain 10 (*EFCAB10*); TAP binding protein-like (*TAPBPL*), tetraspanin 11 (*TSPAN11*), and F-box and leucine-rich repeat protein 14 (*FBXL14*); somatomedin B and thrombospondin type 1 domain containing (*SBSPON*), transmembrane protein 70 (*TMEM70*), and junctophilin 1 (*JPH1*); ER membrane protein complex subunit 8 (*EMC8*); and zinc finger protein 624 (*ZNF624*), centromere protein V (*CENPV*), and unc-51 like autophagy activating kinase 2 (*ULK2*), respectively. On BTA28, the closest genes were coiled-coil serine-rich protein 2 (*CCSER2*), shieldin complex subunit 2 (*SHLD2*), and annexin A8-like protein 1 (*ANXA8L1*), which will be discussed in the section on EBV_{milk}, EBV_{fat}, and EBV_{prot}.

PHTF2 is mainly expressed in muscle and the PHTF family is conserved from *Drosophila* to mammals [76].

EFCAB10 is a complex multidomain protein, with diverse interactions induced by calcium-triggered signals [77], However, no connections with milk properties or composition have been reported.

TAPBPL ameliorated collagen-induced arthritis in mice by reducing autoantibodies [78].

TSPAN11 is a member of the transmembrane superfamily, most of which are cell-surface proteins characterized by four hydrophobic domains. These proteins mediate signal transduction events that play a role in regulating cell development, activation, growth, and motility. A member of this gene family (tetraspanin 4 [*TSPAN4*] on BTA 29) was significantly associated with heifer conception rate in US Holstein cattle [13].

FBXL14 is a member of the F-box protein family which act as protein-ubiquitin ligases. FBXL14 was identified as a novel factor for regulating the radiosensitivity of non-small cell lung cancer in humans [79].

Spermatid perinuclear RNA binding protein (STRBP) involved in spermatogenesis and sperm functions. It plays a role in regulating cell growth. *STRBP* was identified as a possible candidate gene for the examination of bovine male gamete quality [80].

SBSPON encodes a protein coding gene that is predicted to be an extracellular matrix structural constituent and may be involved in calcium signaling and membrane trafficking. When examining miRNA-mRNA pairs, the miR-130a/*SBSPON* was correlated with milk PP in cows [81].

TMEM70 encodes a mitochondrial membrane protein that affects biogenesis of mitochondrial adenosine triphosphate (ATP) synthase and is associated with a rare disorder neonatal mitochondrial encephalo-cardio-myopathy [82] accompanied by elevated lactate and hyperammonemia [83]. However, its effects on cow milk properties have not yet been studied.

JPH1 encodes a protein involved in the formation and maintenance of junctional membrane complexes, which are important for excitation-contraction coupling in skeletal and cardiac muscles. In addition, JPH1 has functional effects on calcium fluxes [84].

EMC8 encodes a protein that is part of the nine-protein transmembrane endoplasmic reticulum chaperon, which is involved in the biogenesis of membrane proteins. The helix in EMC8 is an important interaction site in the formation of voltage-activated calcium ion channels in an ATP-independent manner [85].

ZNF624 encodes a zinc finger protein that may act as a transcription factor and regulate gene expression and was associated with depression scores in humans [86].

CENPV is a histone H3-like protein, which is part of the centromere complex, which is essential for proper chromosome segregation during cell division in mouse oocytes [87].

ULK2 encodes a protein involved in autophagy, a process that degrades and recycles cellular components. Lactoferrin attenuated aflatoxin M1-induced cytotoxicity in human cells by regulating the expression of autophagy factors such as ULK2 [88].

The single SNPs on BTAs 2, 3, 6, 13, 15, 24 and X were located near the genes homeobox D3 (*HOXD3*); RNA binding region (RNP1, RRM) containing 3 (*RNPC3*); heterogeneous nuclear ribonucleoprotein D (*HNRNPD*), heterogeneous nuclear ribonucleoprotein D like (*HNRNPDL*), and kelch like family member 8 (*KLHL8*); patched domain containing 3 (*PTCHD3*); ArfGAP with RhoGAP domain 252C ankyrin repeat and PH domain 1 (*ARAP1*), purinergic receptor P2Y2 (*P2RY2*), prolyl 4-hydroxylase subunit alpha 3 (*P4HA3*), and myosin VIIA (*MYO7A*); zinc finger protein 503 (*ZNF503*) and glyoxalase domain containing 5 (*GLOD5*); GATA binding protein 1 (*GATA1*); histone deacetylase 6 (*HDAC6*); ES cell expressed Ras (*ERAS*); proprotein convertase subtilisin/kexin type 1 inhibitor (*PCSK1N*); translocase of inner mitochondrial membrane 17B (*TIMM17B*); polyglutamine binding protein 1 (*PQBP1*); solute carrier family 35 member A2 (*SLC35A2*); Pim-2 proto-oncogene, serine/threonine kinase (*PIM2*); OTU deubiquitinase 5 (*OTUD5*); potassium voltage-gated channel subfamily D member 1 (*KCND1*); GRIP1 associated protein 1 (*GRIPAP1*); transcription factor binding to IGHM enhancer 3 (*TFE3*); coiled-coil domain containing 120 (*CCDC120*); PRA1 domain family member 2 (*PRAF2*); WD repeat domain 45 (*WDR45*); G-patch domain and KOW motifs (*GPKOW*); proteolipid protein 2 (*PLP2*); prickle planar cell polarity protein 3 (*PRICKLE3*); synaptophysin (*SYP*); calcium voltage-gated channel subunit alpha1 F (*CACNA1F*); coiled-coil domain containing 22 (*CCDC22*); forkhead box P3 (*FOXP3*); protein phosphatase 1 (PP1) regulatory subunit 3F (*PPP1R3F*); PAGE family member 4 (*PAGE4*); ubiquitin specific peptidase 27 X-linked (*USP27X*); and chloride voltage-gated channel 5 (*CLCN5*), respectively. On BTA X, additional genes such as solute carrier

family 38 member 5 (*SLC38A5*), FtsJ RNA methyltransferase homolog 1 (*FTSJ1*), porcupine O-acyltransferase (*PORCN*), emopamil binding protein (sterol isomerase) (*EBP*), TBC1 domain family member 25 (*TBC1D25*), RNA binding motif protein 3 (*RBM3*), WD repeat domain 13 (*WDR13*), and suppressor of variegation 3-9 homolog 1 (*SUV39H1*) were revealed within ±1–3 Mbp.

HOXD3 encodes a transcription factor that is involved in embryonic development and patterning. It belongs to the Hox family of genes, which specify the identity and morphology of body segments and organs. *HOXD3* is expressed in the gut and regulates its maturation and function during the suckling period. Mutations in *HOXD3* impair the assimilation of breast milk and cause growth retardation. Zakany et al. [89] identified HOX3D as the critical component for the gut to maintain milk-digestive competence in mice. HOX3D was also shown to accelerate wound healing in diabetic mice [90].

RNPC3 encodes a protein that is involved in pre-mRNA splicing, especially of U12-type introns, which are processed by the minor spliceosome. It binds to the U12 snRNA and forms part of the U11/U12 small nuclear ribonucleoprotein complex. Mutations in RNPC3 cause hypopituitarism and growth hormone deficiency. It was expressed in pituitary gland, hypothalamus, and ovary in mice [91].

HNRNPD and *HNRNPDL* belong to the subfamily of ubiquitously expressed heterogeneous nuclear ribonucleoproteins (hnRNPs) which are associated with pre-mRNAs in the nucleus and appear to influence pre-mRNA processing and other aspects of mRNA metabolism and transport. *HNRNPDL* was identified as a candidate gene involved in conceptus development in cattle [92].

KLHL8 involved in protein ubiquitination and ubiquitin-dependent protein catabolic process. When examining the effect of SNP-s on EBVs for production and fertility traits in German Holstein cattle, negative correlations was found between yield and fertility traits [93].

The *KLHL8* gene on BTA6 was proposed as a candidate gene for non-return rate in German Holstein heifers. In addition, positive overdominance effects of *KLHL8* were identified in U.S. Holstein cows related to the age at first calving [94].

PTCHD3 was identified as a male germ-cell specific gene, but it was declared to be a non-essential gene in mice [95].

ARAP1 was associated with insulin secretion [96] and was among the genes which alleles were coupled with higher adiposity and lower comorbidities [97].

P2RY2 is considered one of the major purine receptors capable of stimulating chemotaxis via polymorphonuclear neutrophilic leukocytes. P2RY2 was expressed during the peripartal period in cows, increasing from day −12 to day 3 and then decreasing until day 21, suggesting that ATP could play a role in amplifying chemotactic signals [98].

P4HA3 is a component of the prolyl-4-hydroxylases involved in collagen synthesis. In cow, P4HA3 expression was tenfold higher in chorionic gonadotropin-induced follicles than in untreated dominant follicles, small follicles, and the corpus luteum [99].

In mice, the knockdown of *MYO7A* decreased the glucose uptake after oral glucose tolerance test and inhibited the transcription of genes related to insulin and glucose metabolism [100].

In cattle *ZNF503* is highly expressed in lung, adipose tissue, heart and oviduct [101].

GLOD5 encodes a protein with a glyoxalase domain. It is highly expressed in gastrointestinal tract, but no known function is described [102].

GATA1 encodes a transcription factor that regulates the expression of genes involved in erythropoiesis and megakaryopoiesis. It is expressed in the mammary gland, where it may modulate the activity of estrogen receptor alpha and influence the development and function of the mammary gland [103]. GATA1 binds to the promoter of ATP5B [104] and is associated to bull fertility [105].

HDAC6 encodes a histone deacetylase that removes acetyl groups from histone and non-histone proteins and modulates gene expression and protein stability. Its suppression could prevent lipopolysaccharide mediated inflammation [106].

ERAS encodes a protein that activates the phosphatidylinositol 3-kinase signal transduction pathway in undifferentiated stem cells, but is not expressed in differentiated cells. *ERAS* is expressed in the mammary gland, where it may play a role in the tumor-like growth properties of embryonic stem cells [107] and contributes to homeostasis of bovine placenta [108].

PCSK1N encodes a protein that inhibits the proprotein convertases, which are enzymes that process and activate various proteins. PCSK1N may function in the control of the neuroendocrine secretory pathway [109].

TIMM17B encodes a protein that forms an integral component of the mitochondrial translocase TIM23 complex, which mediates the translocation of proteins across the mitochondrial inner membrane. This complex facilitates the transport of mitochondrial proteins from the cytosol across the mitochondrial inner membrane and into the mitochondrion [110]. *TIMM17B* is overexpressed in breast cancer [111].

PQBP1 encodes a protein that interacts with polyglutamine tracts and modulates the transcriptional activity of various nuclear receptors and transcription factors. It can activate transcription directly or via association with the transcription machinery [112]. It plays role in neural functions and developments [113].

SLC35A2 encodes a protein that transports UDP-galactose across the Golgi membrane. *SLC35A2* is expressed in the mammary gland and participates in the lactose synthesis pathway and secretion of milk oligosaccharides in dairy cows [114].

PIM2 encodes a serine/threonine kinase that phosphorylates and regulates various proteins involved in cell survival, proliferation, and differentiation. PIM2 may prevent apoptosis and promote cell survival [115].

OTUD5 encodes a protein that has deubiquitinase activity and suppresses the type I interferon response by cleaving the polyubiquitin chain from an essential type I interferon adaptor protein. OTUD5 may promote the high malignancy of bladder cancer [116].

KCND1 encodes a protein that forms a voltage-gated potassium channel that regulates neuronal excitability, synaptic transmission, heart rate, insulin secretion and smooth muscle contraction [117].

GRIPAP1 encodes a protein that interacts with glutamate receptor interacting protein 1 (GRIP1) and regulates the endosomal recycling of AMPA receptors. *GRIPAP1* is expressed in several tissues, including the mammary gland as well [118] . It has a glutamatergic synapse function and affects behavior in mice [119].

TFE3 encodes a transcription factor that regulates the expression of genes involved in lysosomal biogenesis, autophagy and metabolism of hepatocytes, skeletal muscle cells, adipocytes, and tumor cells [120].

CCDC120 is involved in the regulation of microtubule dynamics and cell cycle progression. It is required for centrosome microtubule anchoring [121] and was associated with high bone mass [122].

PRAF2 is involved in the regulation of protein trafficking and secretion. It was reported to be an oncogene which can promote the proliferation and migration of breast cancer cells [123].

WDR45 encodes a protein involved in autophagy, a process that degrades and recycles cellular components. Mutations in *WDR45* cause a rare neurodegenerative disorder called beta-

propeller protein-associated neurodegeneration, which is characterized by developmental delay, seizures, and brain iron accumulation [124].

GPKOW binds to RNA and regulates its stability and translation. It was associated with age at puberty in Brahman cattle [125].

PLP2 is involved in lipid metabolism and membrane trafficking. PLP2 was found to be associated with mast cell tumors in Holstein cattle [126].

PRICKLE3 encodes a protein involved in the planar cell polarity pathway, which is essential for the polarization of epithelial cells during morphogenetic processes. It plays an important role in pathologies related to the neural and immune systems and cancer development [127].

SYP is involved in synaptic vesicle trafficking and exocytosis. It may play a crucial role in postnatal neurodevelopmental disorders [128]. In cattle, *SYP* was expressed in corpus luteum and showed the highest expression in middle stage of pregnancy, it is important in maintaining functions of corpus luteum [129].

CACNA1F encodes a protein that forms a voltage-gated calcium channel that mediates the influx of calcium ions into excitable cells. It may play a role in alteration of the skeletal muscle and retina functions [130] and global developmental delay [131].

CCDC22 contains a coiled-coil domain that functions in the regulation of nuclear factor kappa-light-chain-enhancer of activated B cells. In humans, *CCDC22* has been identified as a novel candidate gene for syndromic X-linked intellectual disability [132].

FOXP3 encodes a transcription factor that regulates the development and function of T cells, which are important for maintaining immune tolerance and preventing autoimmunity [133]. *FOXP3* is associated with bull fertility traits in cattle [105], and growth traits in sheep [134].

PPP1R3F is a regulatory subunit of protein phosphatase 1, which is involved in glycogen metabolism and insulin signaling. *PPP1R3F* is expressed in tissues related to immune response of organism and may be involved in autoimmune diseases [135].

PAGE4 potentiates the transcriptional activator activity of Jun proto-oncogene AP-1 transcription factor subunit and protects cells from stress-induced apoptosis. *PAGE4* is strongly expressed in prostate and prostate cancer [136].

USP27X has deubiquitinase activity and regulates the stability of various proteins. *USP27X* is expressed in various tissues and may regulate the expression of genes involved in immune responses, and tumorigenesis [137].

CLCN5 forms a voltage-gated chloride channel that mediates the transport of chloride ions across the cell membrane. It is primarily localized to endosomal membranes and may function to facilitate albumin uptake by the renal proximal tubule [138].

SLC38A5 transports amino acids across the cell membrane. It is expressed in the mammary gland, where it plays a role in amino acid uptake [139] and potentially the synthesis and secretion of milk proteins.

FTSJ1 methylates the 2'-O-ribose of nucleotides at positions 32 and 34 of the transfer RNA (tRNA) anticodon loop. Knockdown of *FTSJ1* inhibits breast cancer in humans (Sun et al., 2024) and was associated with intellectual disability in mice [140].

PORCN is involved in the acylation and secretion of Wnt proteins, which are important for cell signaling and development [141]. It has been associated with WNT signaling and overexpressed in trophectoderm compared to inner cell mass of blastocysts in cattle [142].

EBP is involved in the biosynthesis of cholesterol and other sterols [143]. In creep feeding group of Angus x Nelore cattle EBP was upregulated. This group had higher deposition of marbling fat during the postweaning period [144].

TBC1D25 is involved in regulating of membrane trafficking and autophagy. It has also been associated with male infertility [145].

RBM3 is a cold inducible transcription factor. Its expression pattern was under study in in the endometrium and ampulla of cycling heifers [146-147].

WDR13 is involved in transcription regulation and chromatin remodeling [148]. It promoted the differentiation of bovine muscle-derived satellite cells [149].

SUV39H1 is a histone methyltransferase that trimethylates lysine 9 of histone H3, resultg in transcriptional gene silencing [150-151].

EBV_{fat} and EBV_{prot}

Sixteen SNPs on BTA 3, 11, 19, 22, and X were associated with EBV_{fat} and EBV_{prot} (Tables 1, 2). On BTA 3, the region contained cystathionine gamma-lyase (*CTH*). On BTA 11, the regions contained sperm acrosome associated 9 (*SPACA9*), progestagen-associated endometrial protein (*PAEP*), On BTA 19, the regions contained aldehyde dehydrogenase 3 family member A2 (*ALDH3A2*), B9 domain containing 1 (*B9D1*), phosphoribosyl pyrophosphate synthetase associated protein 2 (*PRPSAP2*), myosin XVA (*MYO15A*), and target of myb1 like 2 membrane trafficking protein (*TOM1L2*). On BTA 22, the regions contained deleted in lung and esophageal cancer 1 (*DLEC1*), myeloid differentiation primary response 88 (*MYD88*), solute carrier family 22 member 13 (*SLC22A13*), activin A receptor type 2B (*ACVR2B*), and exo/endonuclease G (*EXOG*); catenin beta 1 (*CTNNB1*), coiled-coil domain containing 13 (*CCDC13*), and atypical chemokine receptor 2 (*ACKR2*); zinc finger protein 852 (*ZNF852*), TatD DNase domain containing 2 (*TATDN2*), RNA pseudouridylate synthase domain containing 3 (*RPUSD3*), tubulin tyrosine ligase like 3 (*TTLL3*), and SET domain containing 5 (*SETD5*); and SLIT-ROBO Rho GTPase activating protein 3 (*SRGAP3*) and oxytocin receptor (*OXTR*). On BTA X, numerous genes were within ±1 Mbp, including neurite extension and migration factor

(*NEXMIF*), ring finger protein LIM domain interacting (*RLIM*), solute carrier family 16 member 2 (*SLC16A2*), zinc finger CCHC-type containing 13 (*ZCCHC13*), cysteine rich hydrophobic domain 1 (*CHIC1*), caudal type homeobox 4 (*CDX4*), uncharacterized protein MGC140080 (*MGC140080*), poly(A) binding protein cytoplasmic 1-like 2A (*PABPC1L2A*), phosphorylase kinase regulatory subunit alpha 1 (*PHKA1*), histone deacetylase 8 (*HDAC8*), Cbp/p300 interacting transactivator with Glu/Asp rich carboxy-terminal domain 1 (*CITED1*), ribosomal protein S4 X-linked (*RPS4X*), ERCC excision repair 6-like spindle assembly checkpoint helicase (*ERCC6L*), peptidylprolyl cis/trans isomerase NIMA-interacting 4 (*PIN4*), NHS like 2 (*NHSL2*), retrotransposon Gag like 5 (*RTL5*), C-X-C motif chemokine receptor 3 (*CXCR3*), germ cell nuclear acidic peptidase (*GCNA*), O-linked N-acetylglucosamine (GlcNAc) transferase (*OGT*), TATA-box binding protein associated factor 1 (*TAF1*), integrin subunit beta 1 binding protein 2 (*ITGB1BP2*), non-POU domain containing octamer binding (*NONO*), zinc finger MYM-type containing 3 (*ZMYM3*), gap junction protein beta 1 (*GJB1*), neuroligin 3 (*NLGN3*), and mediator complex subunit 12 (*MED12*).

CTH is involved in synthesizing of methionine, an essential amino acid for milk protein synthesis. It was found to be downregulated in case of clinical mastitis in Holstein cows [152].

SPACA9 encodes a protein associated with the acrosome of sperm cells and is involved in fertilization. SPACA9 is also found in the lumen of ciliary microtubules, which are important for the movement of cilia and flagella. Cilia and flagella are present in some cells that produce or transport milk, such as mammary gland epithelial cells and lactating alveolar cells [153-154].

PAEP encodes a glycoprotein secreted by the endometrium and found in milk. *PAEP* expression was associated with MY, FY, and PY in Holstein cows [59]. Knutsen et al. [155] have identified SNPs in *PAEP* that lowered the saturated fatty acid ratio (palmitic acid:oleic acid).

ALDH3A2 encodes a protein that catalyzes the oxidation of long-chain aliphatic aldehydes to fatty acids. Mutations in this gene cause Sjogren-Larsson syndrome, a rare disorder characterized by ichthyosis, spasticity, and intellectual disability [156]. Mutations in *ALDH3A2* were associated with fatty acid metabolism and lipid storage in pig [157].

B9D1 encodes for a protein that is part of the B9 complex, which is required for the formation and function of cilia and flagella [158].

PRPSAP2 associates with the enzyme phosphoribosylpyrophosphate synthetase, which is involved in the synthesis of purine and pyrimidine nucleotides, histidine, tryptophan, and nicotinamide adenine dinucleotide. It was associated with osteosarcoma in humans [159].

MYO15A encodes a protein that belongs to the myosin family, which are actin-based motor proteins that generate force and movement. Mutations in this gene cause nonsyndromic deafness [160].

TOM1L2 interacts with target of myb1 membrane trafficking protein protein, which is involved in endosomal trafficking and ubiquitin-dependent protein sorting [161].

DLEC1 is involved in cilia and flagella formation and may act as a tumor suppressor [162]. *DLEC1* was a strongly associated with fertility and reproduction traits in heifers [163].

MYD88 encodes a protein that regulates immune response and inflammation [164]. In circulating leucocytes of cattle, *MYD88* was downregulated during a week after dry-off [165].

SLC22A13 encodes a protein that is a member of the solute carrier family 22, which transports organic ions across membranes. It is maternally expressed in bovine placenta [166].

ACVR2B is a receptor for activin and other members of the transforming growth factor-beta superfamily, which modulate cell growth, differentiation, and apoptosis. It has been associated with heifer fertility traits in *Bos indicus* [167].

EXOG encodes a mitochondrial endo/exonuclease that participates in DNA repair and mitochondrial quality control [168-169].

CTNNB1 encodes a key component of the Wnt signaling pathway, which regulates cell proliferation, differentiation, and survival. *CTNNB1* expression was found to be associated with normal ovarian function in cattle [170]. Farhadian et al. (2018) [171] identified the high connectivity of *CTNNB1* in lactation process.

CCDC13 encodes a protein required for primary cilia formation and promotes the localization of the ciliopathy protein Bardet–Biedl Syndrome 4 to both centriolar satellites and cilia [172]. It was among the proteins associated with endometrial cancer [173].

ACKR2 is an atypical chemokine receptor that controls chemokine levels and localization via high-affinity chemokine binding that is uncoupled from classic ligand-driven signal transduction cascades [174].

ZNF852 is a zinc finger transcription factor and regulates DNA-binding transcription factor activity and has positive regulatory role of ZNF852 in gastric cancer growth and maintenance [175].

TATDN2 is a mitochondrial endo/exonuclease that participates in DNA repair and mitochondrial quality control. It was identified as a therapeutic target in BRCA1 DNA repair-associated (BRCA1)-deficient cancers [176].

RPUSD3 is a mitochondrial RNA pseudouridine synthase that catalyzes the formation of pseudouridine at position 32 in tRNA. It is essential for oxidative phosphorylation pathway for ATP [177].

TTLL3 encodes for a protein that modifies alpha- and beta-tubulin, adding a single glycine on the gamma-carboxyl groups of specific glutamate residues to generate monoglycine side chains within the C-terminal tail of tubulin [178]. It was found to participate in embryonic development, and its abundance decreased from oocyte to blastocyst stage in bovines [179].

SETD5 is a putative methyltransferase and belongs to the epigenetic regulators of gene expression. Mutations in this gene cause intellectual disability and developmental delay [180]. SETD5 in a complex prevents histone acetylation of enhancers for two master adipogenic regulatory genes: CCAAT enhancer binding protein alpha (*CEBPA*) and peroxisome proliferator-activated receptor gamma (*PPARG*) [181].

SRGAP3 encodes a Rho GTPase-activating protein that interacts with the SLIT-ROBO signaling pathway. It is involved in neuronal development and migration [182].

OXTR encodes a receptor for oxytocin, a hormone that stimulates milk ejection and uterine contraction. A novel polymorphism in the OXTR gene affects milk fatty acid composition in Italian Mediterranean river buffalo [183].

NEXMIF is involved in neurite outgrowth and cell-cell adhesion via the N-cadherin signaling pathway. It is also a risk gene for autism spectrum disorder [184].

RLIM encodes an E3 ubiquitin-protein ligase that acts as a negative coregulator for LIM homeodomain transcription factors [185]. It is essential in RLIM-dependent X chromosome inactivation in females [186].

SLC16A2 encodes a transporter of thyroid hormone and facilitates the cellular importation of thyroxine, triiodothyronine, reverse triiodothyronine, and diiodothyronine. Mutations in this gene cause Allan–Herndon–Dudley syndrome, a rare disorder characterized by intellectual disability and abnormal thyroid hormone levels [187]. *SLC16A2* transcript levels did not differ significantly between bovine fetal muscle tissue and muscle fibroblast cultures [188].

ZCCHC13 encodes a zinc finger protein of unknown function that is predicted to be a transcription factor [189]. Its expression correlated with sperm kinematics in cattle [190].

CHIC1 is involved in the regulation of apoptosis and may act as a tumor suppressor [191].

CDX4 encodes a homeobox transcription factor that plays a role in the development of blood cells and the posterior mesoderm [192].

MGC140080 encodes a protein of unknown function that is predicted to be a membrane protein [125].

PABPC1L2A encodes a protein that binds to the poly(A) tail of mRNA molecules and regulates their stability and translation. It is essential for the stability of mRNA in vitro [193].

PHKA1 encodes a subunit of the phosphorylase kinase enzyme, which activates glycogen breakdown in response to hormonal or neural signals. Yang et al. [194] found elevated glycogenolysis with an enhanced phosphorylation of PHKA1 in cattle.

HDAC8 encodes a histone deacetylase that removes acetyl groups from histone proteins and modulates gene expression and chromatin structure [195].

CITED1 encodes a transcriptional coactivator that interacts with several nuclear receptors and transcription factors. A study examining the X chromosome in different cattle breeds identified *CITED1* as an important candidate gene for health traits in the Jersey and Nelore [196]. *CITED1* is also differentially expressed in seminal plasma treated heifers, where the filamentous conceptuses were longer than the control group [92].

RPS4X encodes a ribosomal protein that is part of the small subunit of the ribosome, which is essential for protein synthesis. RPS4X was significantly differentially expressed be-tween bovine fetal muscle tissue and muscle fibroblast cultures [188].

ERCC6L encodes a DNA helicase involved in chromosome separation during cell mitosis. It was found to have a tumor-promoting effect in various cancers [197].

PIN4 encodes a peptidylprolyl cis/trans isomerase that catalyzes the folding of proteins. *PIN4* is ex-pressed in various tissues, including the mammary gland, where it may modulate

the activity of estrogen receptor alpha and influence the development and function of the mammary gland [198].

NHSL2 is involved in the regulation of cell migration and adhesion. In human NHSL2 was related with lower osteogenic and higher adipogenic capacity [78].

The RTL5 gene is derived from a retrovirus that integrated into the mammalian genome. It plays an important role in the innate immune system [199-200].

CXCR3 encodes a chemokine receptor that mediates the chemotaxis of immune cells [201]. Like *CITED1*, *CXCR3* was also identified as an important candidate gene for health traits in Jersey and Nelore cattle [196]. In bovine neonates the lower *CXCR3* expression, among several other down- or upregulated genes, had an immunosuppressive effect [202].

GCNA encodes a protein involved in regulating chromatin structure and gene expression. It is required for the maintenance of the undifferentiated spermatogonia pool and long-term sperm production [203].

OGT encodes a glycosyltransferase that adds a sugar molecule called O-GlcNAc to ser-ine or threonine residues of proteins. *OGT* is essential for mammalian cell viability and may regulate the cell cycle, chromatin structure, and gene expression [204]. It was also found to affect bovine granulosa cell function and glucose metabolism [205].

TAF1 encodes a subunit of the transcription factor IID complex, which is involved in the initiation of RNA polymerase II-mediated transcription [206]. In buffalo, TAF1 expression was significantly higher in the mature compared to the immature Sertoli cells [207].

ITGB1BP2 interacts with integrin beta-1, a cell surface receptor that mediates cell adhesion and signaling [208].

NONO encodes a protein that interacts with integrin beta 1 (ITGB1), a cell surface receptor that medi-ates cell adhesion and signaling [209].

ZMYM3 involved in chromatin structure and gene expression [210]. Its knockout blocks spermatogenesis in mice [211].

GJB1 encodes a protein that forms gap junctions between cells, allowing the exchange of ions and small molecules [212].

NLGN3 was among the genes differentially methylated under MAP infection in ileal and ileal lymph node tissues from cow [213].

MED12 is involved in collagen synthesis as shown in bovine articular chondrocytes [214].

REFERENCES

61. Zhao, G.; Liu, Y.; Niu, Q.; Zheng, X.; Zhang, T.; Wang, Z.; Xu, L.; Zhu, B.; Gao, X.; Zhang, L.; Gao, H.; Li, J.; Xu, L., Runs of homozygosity analysis reveals consensus homozygous regions affecting production traits in Chinese Simmental beef cattle. *BMC Genomics* **2021,** *22* (1), 678, doi:10.1186/s12864-021-07992-6.
62. Iung, L. H. S.; Petrini, J.; Ramirez-Diaz, J.; Salvian, M.; Rovadoscki, G. A.; Pilonetto, F.; Dauria, B. D.; Machado, P. F.; Coutinho, L. L.; Wiggans, G. R.; Mourao, G. B., Genome-wide association study for milk production traits in a Brazilian Holstein population. *J Dairy Sci* **2019,** *102* (6), 5305-5314, doi:10.3168/jds.2018-14811.
63. Yamauchi, K.; Wakabayashi, H.; Shin, K.; Takase, M., Bovine lactoferrin: benefits and mechanism of action against infections. *Biochem Cell Biol* **2006,** *84* (3), 291-6, doi:10.1139/o06-054.
64. Moretti, R.; Soglia, D.; Chessa, S.; Sartore, S.; Finocchiaro, R.; Rasero, R.; Sacchi, P., Identification of SNPs Associated with Somatic Cell Score in Candidate Genes in Italian Holstein Friesian Bulls. *Animals (Basel)* **2021,** *11* (2), doi:10.3390/ani11020366.
65. Tang, J.; Shen, X.; Yang, Y.; Yang, H.; Qi, A.; Yang, S.; Qu, K.; Lan, X.; Huang, B.; Chen, H., Two Different Copy Number Variations of the CLCN2 Gene in Chinese Cattle and Their Association with Growth Traits. *Animals (Basel)* **2021,** *12* (1), doi:10.3390/ani12010041.
66. Cuveillier, C.; Boulan, B.; Ravanello, C.; Denarier, E.; Deloulme, J. C.; Gory-Faure, S.; Delphin, C.; Bosc, C.; Arnal, I.; Andrieux, A., Beyond Neuronal Microtubule Stabilization: MAP6 and CRMPS, Two Converging Stories. *Front Mol Neurosci* **2021,** *14*, 665693, doi:10.3389/fnmol.2021.665693.

67. Linder, M. E.; Deschenes, R. J., Palmitoylation: policing protein stability and traffic. *Nat Rev Mol Cell Biol* **2007,** *8* (1), 74-84, doi:10.1038/nrm2084.
68. Angeli, E.; Rodriguez, F. M.; Rey, F.; Santiago, G.; Matiller, V.; Ortega, H. H.; Hein, G. J., Liver fatty acid metabolism associations with reproductive performance of dairy cattle. *Anim Reprod Sci* **2019,** *208*, 106104, doi:10.1016/j.anireprosci.2019.06.016.
69. TTC14 GeneCards. https://www.genecards.org/cgi-bin/carddisp.pl?gene=TTC14 (accessed on 21 July 2024).
70. Shi, X.; Li, D.; Wang, Y.; Liu, S.; Qin, J.; Wang, J.; Ran, J.; Zhang, Y.; Huang, Q.; Liu, X.; Zhou, J.; Liu, M., Discovery of Centrosomal Protein 70 as an Important Player in the Development and Progression of Breast Cancer. *Am J Pathol* **2017,** *187* (3), 679-688, doi:10.1016/j.ajpath.2016.11.005.
71. Hao, Z.; Zhou, H.; Hickford, J. G. H.; Gong, H.; Wang, J.; Hu, J.; Liu, X.; Li, S.; Zhao, M.; Luo, Y., Identification and characterization of circular RNA in lactating mammary glands from two breeds of sheep with different milk production profiles using RNA-Seq. *Genomics* **2020,** *112* (3), 2186-2193, doi:10.1016/j.ygeno.2019.12.014.
72. Argyriadou, A.; Michailidou, S.; Vouraki, S.; Tsartsianidou, V.; Triantafyllidis, A.; Gelasakis, A.; Banos, G.; Arsenos, G., A genome-wide association study reveals novel SNP markers associated with resilience traits in two Mediterranean dairy sheep breeds. *Front Genet* **2023,** *14*, 1294573, doi:10.3389/fgene.2023.1294573.
73. Liang, X.; Men, Q. L.; Li, Y. W.; Li, H. C.; Chong, T.; Li, Z. L., Silencing of Armadillo Repeat-Containing Protein 8 (ARMc8) Inhibits TGF-beta-Induced EMT in Bladder Carcinoma UMUC3 Cells. *Oncol Res* **2017,** *25* (1), 99-105, doi:10.3727/096504016X14719078133609.
74. Jiang, G.; Zhang, Y.; Zhang, X.; Fan, C.; Wang, L.; Xu, H.; Yu, J.; Wang, E., ARMc8 indicates aggressive colon cancers and promotes invasiveness and migration of colon cancer cells. *Tumour Biol* **2015,** *36* (11), 9005-13, doi:10.1007/s13277-015-3664-z.
75. Suchocki, T.; Egger-Danner, C.; Schwarzenbacher, H.; Szyda, J., Two-stage genome-wide association study for the identification of causal variants underlying hoof disorders in cattle. *J Dairy Sci* **2020,** *103* (5), 4483-4494, doi:10.3168/jds.2019-17542.
76. Manuel, A.; Beaupain, D.; Romeo, P. H.; Raich, N., Molecular characterization of a novel gene family (PHTF) conserved from Drosophila to mammals. *Genomics* **2000,** *64* (2), 216-20, doi:10.1006/geno.1999.6079.
77. Lewit-Bentley, A.; Rety, S., EF-hand calcium-binding proteins. *Curr Opin Struct Biol* **2000,** *10* (6), 637-43, doi:10.1016/s0959-440x(00)00142-1.
78. Zhang, H.; Yang, G.; Li, J.; Xiao, L.; Guo, C.; Wang, Y., Impaired autophagy activity-induced abnormal differentiation of bone marrow stem cells is related to adolescent idiopathic scoliosis osteopenia. *Chin Med J (Engl)* **2023,** *136* (17), 2077-2085, doi:10.1097/CM9.0000000000002165.
79. Cui, Y. H.; Kang, J. H.; Suh, Y.; Zhao, Y.; Yi, J. M.; Bae, I. H.; Lee, H. J.; Park, D. W.; Kim, M. J.; Lee, S. J., Loss of FBXL14 promotes mesenchymal shift and radioresistance of non-small cell lung cancer by TWIST1 stabilization. *Signal Transduct Target Ther* **2021,** *6* (1), 272, doi:10.1038/s41392-021-00599-z.
80. Gilbert, I.; Bissonnette, N.; Boissonneault, G.; Vallee, M.; Robert, C., A molecular analysis of the population of mRNA in bovine spermatozoa. *Reproduction* **2007,** *133* (6), 1073-86, doi:10.1530/REP-06-0292.
81. Ammah, A. A.; Do, D. N.; Bissonnette, N.; Gevry, N.; Ibeagha-Awemu, E. M., Co-Expression Network Analysis Identifies miRNA(-)mRNA Networks Potentially

Regulating Milk Traits and Blood Metabolites. *Int J Mol Sci* **2018,** *19* (9), doi:10.3390/ijms19092500.

82. Sanchez-Caballero, L.; Elurbe, D. M.; Baertling, F.; Guerrero-Castillo, S.; van den Brand, M.; van Strien, J.; van Dam, T. J. P.; Rodenburg, R.; Brandt, U.; Huynen, M. A.; Nijtmans, L. G. J., TMEM70 functions in the assembly of complexes I and V. *Biochim Biophys Acta Bioenerg* **2020,** *1861* (8), 148202, doi:10.1016/j.bbabio.2020.148202.
83. Staretz-Chacham, O.; Wormser, O.; Manor, E.; Birk, O. S.; Ferreira, C. R., TMEM70 deficiency: Novel mutation and hypercitrullinemia during metabolic decompensation. *Am J Med Genet A* **2019,** *179* (7), 1293-1298, doi:10.1002/ajmg.a.61138.
84. Chivet, M.; McCluskey, M.; Nicot, A. S.; Brocard, J.; Beaufils, M.; Giovannini, D.; Giannesini, B.; Poreau, B.; Brocard, J.; Humbert, S.; Saudou, F.; Faure, J.; Marty, I., Huntingtin regulates calcium fluxes in skeletal muscle. *J Gen Physiol* **2023,** *155* (1), doi:10.1085/jgp.202213103.
85. Chen, Z.; Mondal, A.; Abderemane-Ali, F.; Jang, S.; Niranjan, S.; Montano, J. L.; Zaro, B. W.; Minor, D. L., Jr., EMC chaperone-Ca(V) structure reveals an ion channel assembly intermediate. *Nature* **2023,** *619* (7969), 410-419, doi:10.1038/s41586-023-06175-5.
86. Wang, W.; Li, W.; Wu, Y.; Tian, X.; Duan, H.; Li, S.; Tan, Q.; Zhang, D., Genome-wide DNA methylation and gene expression analyses in monozygotic twins identify potential biomarkers of depression. *Transl Psychiatry* **2021,** *11* (1), 416, doi:10.1038/s41398-021-01536-y.
87. Nabi, D.; Drechsler, H.; Pschirer, J.; Korn, F.; Schuler, N.; Diez, S.; Jessberger, R.; Chacon, M., CENP-V is required for proper chromosome segregation through interaction with spindle microtubules in mouse oocytes. *Nat Commun* **2021,** *12* (1), 6547, doi:10.1038/s41467-021-26826-3.
88. Wu, H.; Gao, Y.; Li, S.; Bao, X.; Wang, J.; Zheng, N., Lactoferrin Alleviated AFM1-Induced Apoptosis in Intestinal NCM 460 Cells through the Autophagy Pathway. *Foods* **2021,** *11* (1), doi:10.3390/foods11010023.
89. Zakany, J.; Darbellay, F.; Mascrez, B.; Necsulea, A.; Duboule, D., Control of growth and gut maturation by HoxD genes and the associated lncRNA Haglr. *Proc Natl Acad Sci U S A* **2017,** *114* (44), E9290-E9299, doi:10.1073/pnas.1712511114.
90. Hansen, S. L.; Myers, C. A.; Charboneau, A.; Young, D. M.; Boudreau, N., HoxD3 accelerates wound healing in diabetic mice. *Am J Pathol* **2003,** *163* (6), 2421-31, doi:10.1016/S0002-9440(10)63597-3.
91. Akin, L.; Rizzoti, K.; Gregory, L. C.; Corredor, B.; Le Quesne Stabej, P.; Williams, H.; Buonocore, F.; Mouilleron, S.; Capra, V.; McGlacken-Byrne, S. M.; Martos-Moreno, G. A.; Azmanov, D. N.; Kendirci, M.; Kurtoglu, S.; Suntharalingham, J. P.; Galichet, C.; Gustincich, S.; Tasic, V.; Achermann, J. C.; Accogli, A.; Filipovska, A.; Tuilpakov, A.; Maghnie, M.; Gucev, Z.; Gonen, Z. B.; Perez-Jurado, L. A.; Robinson, I.; Lovell-Badge, R.; Argente, J.; Dattani, M. T., Pathogenic variants in RNPC3 are associated with hypopituitarism and primary ovarian insufficiency. *Genet Med* **2022,** *24* (2), 384-397, doi:10.1016/j.gim.2021.09.019.
92. Mateo-Otero, Y.; Sanchez, J. M.; Recuero, S.; Bages-Arnal, S.; McDonald, M.; Kenny, D. A.; Yeste, M.; Lonergan, P.; Fernandez-Fuertes, B., Effect of Exposure to Seminal Plasma Through Natural Mating in Cattle on Conceptus Length and Gene Expression. *Front Cell Dev Biol* **2020,** *8*, 341, doi:10.3389/fcell.2020.00341.
93. Strucken, E. M.; Bortfeldt, R. H.; Tetens, J.; Thaller, G.; Brockmann, G. A., Genetic effects and correlations between production and fertility traits and their dependency

on the lactation-stage in Holstein Friesians. *BMC Genet* **2012,** *13*, 108, doi:10.1186/1471-2156-13-108.
94. Prakapenka, D.; Liang, Z.; Da, Y., Genome-Wide Association Study of Age at First Calving in U.S. Holstein Cows. *Int J Mol Sci* **2023,** *24* (8), doi:10.3390/ijms24087109.
95. Gonzalez Morales, S. R.; Liu, C.; Blankenship, H.; Zhu, G. Z., Mouse Ptchd3 is a non-essential gene. *Gene X* **2020,** *5*, 100032, doi:10.1016/j.gene.2020.100032.
96. Bailetti, D.; Sentinelli, F.; Prudente, S.; Cimini, F. A.; Barchetta, I.; Totaro, M.; Di Costanzo, A.; Barbonetti, A.; Leonetti, F.; Cavallo, M. G.; Baroni, M. G., Deep Resequencing of 9 Candidate Genes Identifies a Role for ARAP1 and IGF2BP2 in Modulating Insulin Secretion Adjusted for Insulin Resistance in Obese Southern Europeans. *Int J Mol Sci* **2022,** *23* (3), doi:10.3390/ijms23031221.
97. Huang, L. O.; Rauch, A.; Mazzaferro, E.; Preuss, M.; Carobbio, S.; Bayrak, C. S.; Chami, N.; Wang, Z.; Schick, U. M.; Yang, N.; Itan, Y.; Vidal-Puig, A.; den Hoed, M.; Mandrup, S.; Kilpelainen, T. O.; Loos, R. J. F., Genome-wide discovery of genetic loci that uncouple excess adiposity from its comorbidities. *Nat Metab* **2021,** *3* (2), 228-243, doi:10.1038/s42255-021-00346-2.
98. Seo, J.; Osorio, J. S.; Loor, J. J., Purinergic signaling gene network expression in bovine polymorphonuclear neutrophils during the peripartal period. *J Dairy Sci* **2013,** *96* (12), 7675-83, doi:10.3168/jds.2013-6952.
99. Lussier, J. G.; Diouf, M. N.; Levesque, V.; Sirois, J.; Ndiaye, K., Gene expression profiling of upregulated mRNAs in granulosa cells of bovine ovulatory follicles following stimulation with hCG. *Reprod Biol Endocrinol* **2017,** *15* (1), 88, doi:10.1186/s12958-017-0306-x.
100. Wu, Y.; Zhang, C.; Duan, S.; Li, Y.; Lu, L.; Bajpai, A.; Yang, C.; Mi, J.; Tian, G.; Xu, F.; Qi, D.; Xu, Z.; Chi, X. D., TEAD1, MYO7A and NDUFC2 are novel functional genes associated with glucose metabolism in BXD recombinant inbred population. *Diabetes Obes Metab* **2024,** *26* (5), 1775-1788, doi:10.1111/dom.15491.
101. The cattle Genotype-Tissue Expression atlas. https://cgtex.roslin.ed.ac.uk/search/ (accessed on 21 July 2024).
102. Farrera, D. O.; Galligan, J. J., The Human Glyoxalase Gene Family in Health and Disease. *Chem Res Toxicol* **2022,** *35* (10), 1766-1776, doi:10.1021/acs.chemrestox.2c00182.
103. Ferreira, R.; Ohneda, K.; Yamamoto, M.; Philipsen, S., GATA1 function, a paradigm for transcription factors in hematopoiesis. *Mol Cell Biol* **2005,** *25* (4), 1215-27, doi:10.1128/MCB.25.4.1215-1227.2005.
104. Zhao, Z.; Raza, S. H. A.; Luo, Y.; Wang, J.; Liu, X.; Li, S.; Shi, B.; Hu, J., Characterization of the promoter region of bovine ATP5B: roles of MyoD and GATA1 in the regulation of basal transcription. *Anim Biotechnol* **2022,** *33* (4), 757-764, doi:10.1080/10495398.2020.1837848.
105. Tan, W. L. A.; Hudson, N. J.; Porto Neto, L. R.; Reverter, A.; Afonso, J.; Fortes, M. R. S., An association weight matrix identified biological pathways associated with bull fertility traits in a multi-breed population. *Anim Genet* **2024**, doi:10.1111/age.13431.
106. Wang, J.; Zhao, L.; Wei, Z.; Zhang, X.; Wang, Y.; Li, F.; Fu, Y.; Liu, B., Inhibition of histone deacetylase reduces lipopolysaccharide-induced-inflammation in primary mammary epithelial cells by regulating ROS-NF-small ka, CyrillicB signaling pathways. *Int Immunopharmacol* **2018,** *56*, 230-234, doi:10.1016/j.intimp.2018.01.039.

107. Suarez-Cabrera, C.; Ojeda-Perez, I.; Sanchez-Baltasar, R.; Page, A.; Bravo, A.; Navarro, M.; Ramirez, A., ERAS, a Member of the Ras Superfamily, Acts as an Oncoprotein in the Mammary Gland. *Cancers (Basel)* **2021,** *13* (21), doi:10.3390/cancers13215588.
108. Roperto, S.; Russo, V.; Urraro, C.; Restucci, B.; Corrado, F.; De Falco, F.; Roperto, F., ERas is constitutively expressed in full term placenta of pregnant cows. *Theriogenology* **2017,** *103*, 162-168, doi:10.1016/j.theriogenology.2017.07.047.
109. PCSK1N GeneCards. (https://www.genecards.org/cgi-bin/carddisp.pl?gene=PCSK1N (accessed on 21 July 2024).
110. TIMM17B National Library of Medicine. https://www.ncbi.nlm.nih.gov/gene/10245 (accessed on 21 July 2024).
111. Mingting, D.; Yun, R.; Jiwen, Z.; Tingting, Z.; Yanhong, W.; Hongyan, J., High Expression of TIMM17B Is a Potential Diagnostic and Prognostic Marker of Breast Cancer. *Cell Mol Biol (Noisy-le-grand)* **2023,** *69* (3), 169-176, doi:10.14715/cmb/2023.69.3.25.
112. Tanaka, H.; Okazawa, H., PQBP1: The Key to Intellectual Disability, Neurodegenerative Diseases, and Innate Immunity. *Int J Mol Sci* **2022,** *23* (11), doi:10.3390/ijms23116227.
113. Cheng, S.; Liu, X.; Yuan, L.; Wang, N.; Zhang, Z. C.; Han, J., The role of PQBP1 in neural development and function. *Biochem Soc Trans* **2023,** *51* (1), 363-372, doi:10.1042/BST20220920.
114. Lin, Y.; Sun, X.; Hou, X.; Qu, B.; Gao, X.; Li, Q., Effects of glucose on lactose synthesis in mammary epithelial cells from dairy cow. *BMC Vet Res* **2016,** *12*, 81, doi:10.1186/s12917-016-0704-x.
115. Wang, Y.; Xiu, J.; Ren, C.; Yu, Z., Protein kinase PIM2: A simple PIM family kinase with complex functions in cancer metabolism and therapeutics. *J Cancer* **2021,** *12* (9), 2570-2581, doi:10.7150/jca.53134.
116. Hou, T.; Dan, W.; Liu, T.; Liu, B.; Wei, Y.; Yue, C.; Que, T.; Ma, B.; Lei, Y.; Wang, Z.; Zeng, J.; Fan, Y.; Li, L., Deubiquitinase OTUD5 modulates mTORC1 signaling to promote bladder cancer progression. *Cell Death Dis* **2022,** *13* (9), 778, doi:10.1038/s41419-022-05128-6.
117. Isbrandt, D.; Leicher, T.; Waldschutz, R.; Zhu, X.; Luhmann, U.; Michel, U.; Sauter, K.; Pongs, O., Gene structures and expression profiles of three human KCND (Kv4) potassium channels mediating A-type currents I(TO) and I(SA). *Genomics* **2000,** *64* (2), 144-54, doi:10.1006/geno.2000.6117.
118. GRIPAP1 The Human Protein Atlas. https://www.proteinatlas.org/ENSG00000068400-GRIPAP1/tissue (accessed on 21 July 2024).
119. Chiu, S. L.; Diering, G. H.; Ye, B.; Takamiya, K.; Chen, C. M.; Jiang, Y.; Niranjan, T.; Schwartz, C. E.; Wang, T.; Huganir, R. L., GRASP1 Regulates Synaptic Plasticity and Learning through Endosomal Recycling of AMPA Receptors. *Neuron* **2017,** *93* (6), 1405-1419 e8, doi:10.1016/j.neuron.2017.02.031.
120. Li, X.; Chen, Y.; Gong, S.; Chen, H.; Liu, H.; Li, X.; Hao, J., Emerging roles of TFE3 in metabolic regulation. *Cell Death Discov* **2023,** *9* (1), 93, doi:10.1038/s41420-023-01395-0.
121. Huang, N.; Xia, Y.; Zhang, D.; Wang, S.; Bao, Y.; He, R.; Teng, J.; Chen, J., Hierarchical assembly of centriole subdistal appendages via centrosome binding proteins CCDC120 and CCDC68. *Nat Commun* **2017,** *8*, 15057, doi:10.1038/ncomms15057.
122. Tuysuz, B.; Usluer, E.; Uludag Alkaya, D.; Ocak, S.; Saygili, S.; Seker, A.; Apak, H., The molecular spectrum of Turkish osteopetrosis and related osteoclast disorders with

natural history, including a candidate gene, CCDC120. *Bone* **2023,** *177*, 116897, doi:10.1016/j.bone.2023.116897.

123. Wang, Y.; Zhao, Z.; Jiao, W.; Yin, Z.; Zhao, W.; Bo, H.; Bi, Z.; Dong, B.; Chen, B.; Wang, Z., PRAF2 is an oncogene acting to promote the proliferation and invasion of breast cancer cells. *Exp Ther Med* **2022,** *24* (6), 738, doi:10.3892/etm.2022.11674.
124. Cong, Y.; So, V.; Tijssen, M. A. J.; Verbeek, D. S.; Reggiori, F.; Mauthe, M., WDR45, one gene associated with multiple neurodevelopmental disorders. *Autophagy* **2021,** *17* (12), 3908-3923, doi:10.1080/15548627.2021.1899669.
125. Fortes, M. R. S.; Lehnert, S. A.; Bolormaa, S.; Reich, C.; Fordyce, G.; Corbet, N. J.; Whan, V.; Hawken, R. J.; Reverter, A., Finding genes for economically important traits: Brahman cattle puberty. *Animal Production Science* **2012,** *52* (3), 143-150, doi:https://doi.org/10.1071/AN11165.
126. Jacinto, J. G. P.; Muscatello, L. V.; Hafliger, I. M.; Benazzi, C.; Bolcato, M.; Gentile, A.; Drogemuller, C., A Missense Variant in PLP2 in Holstein Cattle with X-Linked Congenital Mast Cell Tumor. *Animals (Basel)* **2022,** *12* (18), doi:10.3390/ani12182329.
127. Radaszkiewicz, K. A.; Sulcova, M.; Kohoutkova, E.; Harnos, J., The role of prickle proteins in vertebrate development and pathology. *Mol Cell Biochem* **2024,** *479* (5), 1199-1221, doi:10.1007/s11010-023-04787-z.
128. Aizawa, S.; Yamamuro, Y., Possible involvement of DNA methylation in hippocampal synaptophysin gene expression during postnatal development of mice. *Neurochem Int* **2020,** *132*, 104587, doi:10.1016/j.neuint.2019.104587.
129. Zhang, W.; Chen, S.; Wang, Z.; Tang, C.; Meng, X.; Li, F.; Zhao, S., Expression of synaptophysin and its mRNA in bovine corpus lutea during different stages of pregnancy. *Res Vet Sci* **2013,** *94* (3), 449-52, doi:10.1016/j.rvsc.2012.10.024.
130. An, J.; Zhang, L.; Jiao, B.; Lu, F.; Xia, F.; Yu, Z.; Zhang, Z., Cacna1f gene decreased contractility of skeletal muscle in rat model with congenital stationary night blindness. *Gene* **2015,** *562* (2), 210-9, doi:10.1016/j.gene.2015.02.073.
131. Kessi, M.; Chen, B.; Peng, J.; Yan, F.; Yang, L.; Yin, F., Calcium channelopathies and intellectual disability: a systematic review. *Orphanet J Rare Dis* **2021,** *16* (1), 219, doi:10.1186/s13023-021-01850-0.
132. Kolanczyk, M.; Krawitz, P.; Hecht, J.; Hupalowska, A.; Miaczynska, M.; Marschner, K.; Schlack, C.; Emmerich, D.; Kobus, K.; Kornak, U.; Robinson, P. N.; Plecko, B.; Grangl, G.; Uhrig, S.; Mundlos, S.; Horn, D., Missense variant in CCDC22 causes X-linked recessive intellectual disability with features of Ritscher-Schinzel/3C syndrome. *Eur J Hum Genet* **2015,** *23* (5), 720, doi:10.1038/ejhg.2014.278.
133. Pacheco-Gonzalez, R. M.; Avila, C.; Davila, I.; Garcia-Sanchez, A.; Hernandez-Hernandez, L.; Benito-Pescador, D.; Torres, R.; Prieto-Matos, P.; Isidoro-Garcia, M.; Lorente, F.; Sanz, C., Analysis of FOXP3 gene in children with allergy and autoimmune diseases. *Allergol Immunopathol (Madr)* **2016,** *44* (1), 32-40, doi:10.1016/j.aller.2015.01.012.
134. Ibrahim, A. H. M.; Megaley, A. F. M.; Sallam, A. M. A., Variation in the ovine FOXP3 gene and its effect on growth traits in Egyptian Barki sheep. *Anim Biotechnol* **2023,** *34* (3), 679-685, doi:10.1080/10495398.2021.1996387.
135. Chang, D.; Gao, F.; Slavney, A.; Ma, L.; Waldman, Y. Y.; Sams, A. J.; Billing-Ross, P.; Madar, A.; Spritz, R.; Keinan, A., Accounting for eXentricities: analysis of the X chromosome in GWAS reveals X-linked genes implicated in autoimmune diseases. *PLoS One* **2014,** *9* (12), e113684, doi:10.1371/journal.pone.0113684.

136. Kulkarni, P.; Dunker, A. K.; Weninger, K.; Orban, J., Prostate-associated gene 4 (PAGE4), an intrinsically disordered cancer/testis antigen, is a novel therapeutic target for prostate cancer. *Asian J Androl* **2016,** *18* (5), 695-703, doi:10.4103/1008-682X.181818.
137. Tao, X.; Chu, B.; Xin, D.; Li, L.; Sun, Q., USP27X negatively regulates antiviral signaling by deubiquitinating RIG-I. *PLoS Pathog* **2020,** *16* (2), e1008293, doi:10.1371/journal.ppat.1008293.
138. Hayward, S.; Norton, J.; Bownass, L.; Platt, C.; Genomics England Research, C.; Campbell, H.; Watson, E.; Forrester, N.; Smithson, S.; Menon, A., A novel likely pathogenic CLCN5 variant in Dent's disease. *BMC Nephrol* **2023,** *24* (1), 256, doi:10.1186/s12882-023-03292-1.
139. Wang, H.; Shen, Q.; Ye, L. H.; Ye, J., MED12 mutations in human diseases. *Protein Cell* **2013,** *4* (9), 643-6, doi:10.1007/s13238-013-3048-3.
140. Nagayoshi, Y.; Chujo, T.; Hirata, S.; Nakatsuka, H.; Chen, C. W.; Takakura, M.; Miyauchi, K.; Ikeuchi, Y.; Carlyle, B. C.; Kitchen, R. R.; Suzuki, T.; Katsuoka, F.; Yamamoto, M.; Goto, Y.; Tanaka, M.; Natsume, K.; Nairn, A. C.; Suzuki, T.; Tomizawa, K.; Wei, F. Y., Loss of Ftsj1 perturbs codon-specific translation efficiency in the brain and is associated with X-linked intellectual disability. *Sci Adv* **2021,** *7* (13), doi:10.1126/sciadv.abf3072.
141. Tuladhar, R.; Yarravarapu, N.; Ma, Y.; Zhang, C.; Herbert, J.; Kim, J.; Chen, C.; Lum, L., Stereoselective fatty acylation is essential for the release of lipidated WNT proteins from the acyltransferase Porcupine (PORCN). *J Biol Chem* **2019,** *294* (16), 6273-6282, doi:10.1074/jbc.RA118.007268.
142. Tribulo, P.; Moss, J. I.; Ozawa, M.; Jiang, Z.; Tian, X. C.; Hansen, P. J., WNT regulation of embryonic development likely involves pathways independent of nuclear CTNNB1. *Reproduction* **2017,** *153* (4), 405-419, doi:10.1530/REP-16-0610.
143. Jiang, L.; Niu, W.; Zheng, Q.; Meng, G.; Chen, X.; Zhang, M.; Deng, G.; Mao, Q.; Wang, L., Identification of an Autoantibody Against ErbB-3-Binding Protein-1 in the Sera of Patients With Chronic Hepatitis B Virus Infection. *Front Immunol* **2021,** *12*, 640335, doi:10.3389/fimmu.2021.640335.
144. Ramirez-Zamudio, G. D.; Ganga, M. J. G.; Pereira, G. L.; Nociti, R. P.; Chiaratti, M. R.; Cooke, R. F.; Chardulo, L. A. L.; Baldassini, W. A.; Machado-Neto, O. R.; Curi, R. A., Effect of Cow-Calf Supplementation on Gene Expression, Processes, and Pathways Related to Adipogenesis and Lipogenesis in Longissimus thoracis Muscle of F1 Angus x Nellore Cattle at Weaning. *Metabolites* **2023,** *13* (2), doi:10.3390/metabo13020160.
145. Nawaz, S.; Hussain, S.; Basit, S.; Ahmad, W., First evidence of involvement of TBC1D25 in causing human male infertility. *Eur J Med Genet* **2021,** *64* (2), 104142, doi:10.1016/j.ejmg.2021.104142.
146. Gardela, J.; Ruiz-Conca, M.; Olvera-Maneu, S.; Lopez-Bejar, M.; Alvarez-Rodriguez, M., The mRNA expression of the three major described cold-inducible proteins, including CIRBP, differs in the bovine endometrium and ampulla during the estrous cycle. *Res Vet Sci* **2022,** *152*, 181-189, doi:10.1016/j.rvsc.2022.08.006.
147. Wellmann, S.; Truss, M.; Bruder, E.; Tornillo, L.; Zelmer, A.; Seeger, K.; Buhrer, C., The RNA-binding protein RBM3 is required for cell proliferation and protects against serum deprivation-induced cell death. *Pediatr Res* **2010,** *67* (1), 35-41, doi:10.1203/PDR.0b013e3181c13326.

148. Rzonca-Niewczas, S.; Wierzba, J.; Kaczorowska, E.; Poryszewska, M.; Kosinska, J.; Stawinski, P.; Ploski, R.; Bal, J., WDR13: A Novel Gene Implicated in Non-Syndromic Intellectual Disability. *Genes (Basel)* **2021,** *12* (12), doi:10.3390/genes12121911.
149. Fu, Y.; Li, S.; Tong, H.; Li, S.; Yan, Y., WDR13 promotes the differentiation of bovine skeletal muscle-derived satellite cells by affecting PI3K/AKT signaling. *Cell Biol Int* **2019,** *43* (7), 799-808, doi:10.1002/cbin.11160.
150. Chen, T. T.; Wu, S. M.; Ho, S. C.; Chuang, H. C.; Liu, C. Y.; Chan, Y. F.; Kuo, L. W.; Feng, P. H.; Liu, W. T.; Chen, K. Y.; Hsiao, T. C.; Juang, J. N.; Lee, K. Y., SUV39H1 Reduction Is Implicated in Abnormal Inflammation in COPD. *Sci Rep* **2017,** *7*, 46667, doi:10.1038/srep46667.
151. Weirich, S.; Khella, M. S.; Jeltsch, A., Structure, Activity and Function of the Suv39h1 and Suv39h2 Protein Lysine Methyltransferases. *Life (Basel)* **2021,** *11* (7), doi:10.3390/life11070703.
152. Zhang, B.; Lin, T.; Bai, X.; An, X.; Dai, L.; Shi, J.; Zhang, Y.; Zhao, X.; Zhang, Q., Sulfur Amino Acid Metabolism and the Role of Endogenous Cystathionine-gamma-lyase/H(2)S in Holstein Cows with Clinical Mastitis. *Animals (Basel)* **2022,** *12* (11), doi:10.3390/ani12111451.
153. Tijjani, A.; Utsunomiya, Y. T.; Ezekwe, A. G.; Nashiru, O.; Hanotte, O., Genome Sequence Analysis Reveals Selection Signatures in Endangered Trypanotolerant West African Muturu Cattle. *Front Genet* **2019,** *10*, 442, doi:10.3389/fgene.2019.00442.
154. Gui, M.; Croft, J. T.; Zabeo, D.; Acharya, V.; Kollman, J. M.; Burgoyne, T.; Hoog, J. L.; Brown, A., SPACA9 is a lumenal protein of human ciliary singlet and doublet microtubules. *Proc Natl Acad Sci U S A* **2022,** *119* (41), e2207605119, doi:10.1073/pnas.2207605119.
155. Knutsen, T. M.; Olsen, H. G.; Ketto, I. A.; Sundsaasen, K. K.; Kohler, A.; Tafintseva, V.; Svendsen, M.; Kent, M. P.; Lien, S., Genetic variants associated with two major bovine milk fatty acids offer opportunities to breed for altered milk fat composition. *Genet Sel Evol* **2022,** *54* (1), 35, doi:10.1186/s12711-022-00731-9.
156. Yin, Z.; Wu, D.; Shi, J.; Wei, X.; Jin, N.; Lu, X.; Ren, X., Identification of ALDH3A2 as a novel prognostic biomarker in gastric adenocarcinoma using integrated bioinformatics analysis. *BMC Cancer* **2020,** *20* (1), 1062, doi:10.1186/s12885-020-07493-x.
157. Piorkowska, K.; Zukowski, K.; Ropka-Molik, K.; Tyra, M., Detection of genetic variants between different Polish Landrace and Pulawska pigs by means of RNA-seq analysis. *Anim Genet* **2018,** *49* (3), 215-225, doi:10.1111/age.12654.
158. Okazaki, M.; Kobayashi, T.; Chiba, S.; Takei, R.; Liang, L.; Nakayama, K.; Katoh, Y., Formation of the B9-domain protein complex MKS1-B9D2-B9D1 is essential as a diffusion barrier for ciliary membrane proteins. *Mol Biol Cell* **2020,** *31* (20), 2259-2268, doi:10.1091/mbc.E20-03-0208.
159. Both, J.; Wu, T.; Bras, J.; Schaap, G. R.; Baas, F.; Hulsebos, T. J., Identification of novel candidate oncogenes in chromosome region 17p11.2-p12 in human osteosarcoma. *PLoS One* **2012,** *7* (1), e30907, doi:10.1371/journal.pone.0030907.
160. Zhang, J.; Guan, J.; Wang, H.; Yin, L.; Wang, D.; Zhao, L.; Zhou, H.; Wang, Q., Genotype-phenotype correlation analysis of MYO15A variants in autosomal recessive non-syndromic hearing loss. *BMC Med Genet* **2019,** *20* (1), 60, doi:10.1186/s12881-019-0790-2.

161. TOM1L2 GeneCards. https://www.genecards.org/cgi-bin/carddisp.pl?gene=TOM1L2 (accessed on 21 July 2024).
162. DLEC1 National Library of Medicine. https://www.ncbi.nlm.nih.gov/gene/9940 (accessed on 21 July 2024).
163. Chen, S. Y.; Schenkel, F. S.; Melo, A. L. P.; Oliveira, H. R.; Pedrosa, V. B.; Araujo, A. C.; Melka, M. G.; Brito, L. F., Identifying pleiotropic variants and candidate genes for fertility and reproduction traits in Holstein cattle via association studies based on imputed whole-genome sequence genotypes. *BMC Genomics* **2022,** *23* (1), 331, doi:10.1186/s12864-022-08555-z.
164. Biswas, C., in Immunity and inflammation in health and disease: emerging roles of nutraceuticals and functional foods in immune support; edited by Chatterjee S, Jungraithmayr W and Bagchi D. London, United Kingdom; Cambridge, MA, United States: Elsevier/Academic Press. **2018**.
165. Cattaneo, L.; Mezzetti, M.; Lopreiato, V.; Piccioli-Cappelli, F.; Trevisi, E.; Minuti, A., Gene network expression of whole blood leukocytes in dairy cows with different milk yield at dry-off. *PLoS One* **2021,** *16* (12), e0260745, doi:10.1371/journal.pone.0260745.
166. Liu, X.; Huo, H.; Jin, L.; Dong, Y.; Li, D.; Zhang, C.; Li, S., Genomic imprinting of the IGF2R/AIR locus is conserved between bovines and mice. *Theriogenology* **2022,** *180*, 121-129, doi:10.1016/j.theriogenology.2021.12.013.
167. Tahir, M. S.; Porto-Neto, L. R.; Gondro, C.; Shittu, O. B.; Wockner, K.; Tan, A. W. L.; Smith, H. R.; Gouveia, G. C.; Kour, J.; Fortes, M. R. S., Meta-Analysis of Heifer Traits Identified Reproductive Pathways in Bos indicus Cattle. *Genes (Basel)* **2021,** *12* (5), doi:10.3390/genes12050768.
168. Szymanski, M. R.; Yu, W.; Gmyrek, A. M.; White, M. A.; Molineux, I. J.; Lee, J. C.; Yin, Y. W., A domain in human EXOG converts apoptotic endonuclease to DNA-repair exonuclease. *Nat Commun* **2017,** *8*, 14959, doi:10.1038/ncomms14959.
169. Wu, C. C.; Lin, J. L. J.; Yang-Yen, H. F.; Yuan, H. S., A unique exonuclease ExoG cleaves between RNA and DNA in mitochondrial DNA replication. *Nucleic Acids Res* **2019,** *47* (10), 5405-5419, doi:10.1093/nar/gkz241.
170. Gomez, B. I.; Aloqaily, B. H.; Gifford, C. A.; Hallford, D. M.; Hernandez Gifford, J. A., ASAS-SSR Triennial Reproduction Symposium: Looking Back and Moving Forward-How Reproductive Physiology has Evolved: WNTs role in bovine folliculogenesis and estrogen production. *J Anim Sci* **2018,** *96* (7), 2977-2986, doi:10.1093/jas/sky135.
171. Farhadian, M.; Rafat, S. A.; Hasanpur, K.; Ebrahimi, M.; Ebrahimie, E., Cross-Species Meta-Analysis of Transcriptomic Data in Combination With Supervised Machine Learning Models Identifies the Common Gene Signature of Lactation Process. *Front Genet* **2018,** *9*, 235, doi:10.3389/fgene.2018.00235.
172. Staples, C. J.; Myers, K. N.; Beveridge, R. D.; Patil, A. A.; Howard, A. E.; Barone, G.; Lee, A. J.; Swanton, C.; Howell, M.; Maslen, S.; Skehel, J. M.; Boulton, S. J.; Collis, S. J., Ccdc13 is a novel human centriolar satellite protein required for ciliogenesis and genome stability. *J Cell Sci* **2014,** *127* (Pt 13), 2910-9, doi:10.1242/jcs.147785.
173. Taylor, A. H.; Konje, J. C.; Ayakannu, T., Identification of Potentially Novel Molecular Targets of Endometrial Cancer Using a Non-Biased Proteomic Approach. *Cancers (Basel)* **2023,** *15* (18), doi:10.3390/cancers15184665.

174. Bonavita, O.; Mollica Poeta, V.; Setten, E.; Massara, M.; Bonecchi, R., ACKR2: An Atypical Chemokine Receptor Regulating Lymphatic Biology. *Front Immunol* **2016,** *7*, 691, doi:10.3389/fimmu.2016.00691.
175. Ke, C.; Zhou, H.; Jiang, B.; Xie, X., Zinc finger protein 852 is essential for the proliferation, drug sensitivity, and self-renewal of gastric cancer cells. *Cell Biol Int* **2022,** *46* (4), 579-587, doi:10.1002/cbin.11754.
176. Jaiswal, A. S.; Dutta, A.; Srinivasan, G.; Yuan, Y.; Zhou, D.; Shaheen, M.; Sadideen, D. T.; Kirby, A.; Williamson, E. A.; Gupta, Y. K.; Olsen, S. K.; Xu, M.; Loranc, E.; Mukhopadhyay, P.; Pertsemlidis, A.; Bishop, A. J. R.; Sung, P.; Nickoloff, J. A.; Hromas, R., TATDN2 resolution of R-loops is required for survival of BRCA1-mutant cancer cells. *Nucleic Acids Res* **2023,** *51* (22), 12224-12241, doi:10.1093/nar/gkad952.
177. Arroyo, J. D.; Jourdain, A. A.; Calvo, S. E.; Ballarano, C. A.; Doench, J. G.; Root, D. E.; Mootha, V. K., A Genome-wide CRISPR Death Screen Identifies Genes Essential for Oxidative Phosphorylation. *Cell Metab* **2016,** *24* (6), 875-885, doi:10.1016/j.cmet.2016.08.017.
178. Wloga, D.; Webster, D. M.; Rogowski, K.; Bre, M. H.; Levilliers, N.; Jerka-Dziadosz, M.; Janke, C.; Dougan, S. T.; Gaertig, J., TTLL3 Is a tubulin glycine ligase that regulates the assembly of cilia. *Dev Cell* **2009,** *16* (6), 867-76, doi:10.1016/j.devcel.2009.04.008.
179. Ortega, M. S.; Kurian, J. J.; McKenna, R.; Hansen, P. J., Characteristics of candidate genes associated with embryonic development in the cow: Evidence for a role for WBP1 in development to the blastocyst stage. *PLoS One* **2017,** *12* (5), e0178041, doi:10.1371/journal.pone.0178041.
180. Kuechler, A.; Zink, A. M.; Wieland, T.; Ludecke, H. J.; Cremer, K.; Salviati, L.; Magini, P.; Najafi, K.; Zweier, C.; Czeschik, J. C.; Aretz, S.; Endele, S.; Tamburrino, F.; Pinato, C.; Clementi, M.; Gundlach, J.; Maylahn, C.; Mazzanti, L.; Wohlleber, E.; Schwarzmayr, T.; Kariminejad, R.; Schlessinger, A.; Wieczorek, D.; Strom, T. M.; Novarino, G.; Engels, H., Loss-of-function variants of SETD5 cause intellectual disability and the core phenotype of microdeletion 3p25.3 syndrome. *Eur J Hum Genet* **2015,** *23* (6), 753-60, doi:10.1038/ejhg.2014.165.
181. Matsumura, Y.; Ito, R.; Yajima, A.; Yamaguchi, R.; Tanaka, T.; Kawamura, T.; Magoori, K.; Abe, Y.; Uchida, A.; Yoneshiro, T.; Hirakawa, H.; Zhang, J.; Arai, M.; Yang, C.; Yang, G.; Takahashi, H.; Fujihashi, H.; Nakaki, R.; Yamamoto, S.; Ota, S.; Tsutsumi, S.; Inoue, S. I.; Kimura, H.; Wada, Y.; Kodama, T.; Inagaki, T.; Osborne, T. F.; Aburatani, H.; Node, K.; Sakai, J., Spatiotemporal dynamics of SETD5-containing NCoR-HDAC3 complex determines enhancer activation for adipogenesis. *Nat Commun* **2021,** *12* (1), 7045, doi:10.1038/s41467-021-27321-5.
182. Bacon, C.; Endris, V.; Rappold, G. A., The cellular function of srGAP3 and its role in neuronal morphogenesis. *Mech Dev* **2013,** *130* (6-8), 391-5, doi:10.1016/j.mod.2012.10.005.
183. Cosenza, G.; Macciotta, N. P. P.; Nudda, A.; Coletta, A.; Ramunno, L.; Pauciullo, A., A novel polymorphism in the oxytocin receptor encoding gene (OXTR) affects milk fatty acid composition in Italian Mediterranean river buffalo. *J Dairy Res* **2017,** *84* (2), 170-180, doi:10.1017/S0022029917000127.
184. Stamberger, H.; Hammer, T. B.; Gardella, E.; Vlaskamp, D. R. M.; Bertelsen, B.; Mandelstam, S.; de Lange, I.; Zhang, J.; Myers, C. T.; Fenger, C.; Afawi, Z.; Almanza Fuerte, E. P.; Andrade, D. M.; Balcik, Y.; Ben Zeev, B.; Bennett, M. F.; Berkovic, S. F.; Isidor, B.; Bouman, A.; Brilstra, E.; Busk, O. L.; Cairns, A.; Caumes, R.; Chatron, N.;

Dale, R. C.; de Geus, C.; Edery, P.; Gill, D.; Granild-Jensen, J. B.; Gunderson, L.; Gunning, B.; Heimer, G.; Helle, J. R.; Hildebrand, M. S.; Hollingsworth, G.; Kharytonov, V.; Klee, E. W.; Koeleman, B. P. C.; Koolen, D. A.; Korff, C.; Kury, S.; Lesca, G.; Lev, D.; Leventer, R. J.; Mackay, M. T.; Macke, E. L.; McEntagart, M.; Mohammad, S. S.; Monin, P.; Montomoli, M.; Morava, E.; Moutton, S.; Muir, A. M.; Parrini, E.; Procopis, P.; Ranza, E.; Reed, L.; Reif, P. S.; Rosenow, F.; Rossi, M.; Sadleir, L. G.; Sadoway, T.; Schelhaas, H. J.; Schneider, A. L.; Shah, K.; Shalev, R.; Sisodiya, S. M.; Smol, T.; Stumpel, C.; Stuurman, K.; Symonds, J. D.; Mau-Them, F. T.; Verbeek, N.; Verhoeven, J. S.; Wallace, G.; Yosovich, K.; Zarate, Y. A.; Zerem, A.; Zuberi, S. M.; Guerrini, R.; Mefford, H. C.; Patel, C.; Zhang, Y. H.; Moller, R. S.; Scheffer, I. E., NEXMIF encephalopathy: an X-linked disorder with male and female phenotypic patterns. *Genet Med* **2021,** *23* (2), 363-373, doi:10.1038/s41436-020-00988-9.

185. Frints, S. G. M.; Ozanturk, A.; Rodriguez Criado, G.; Grasshoff, U.; de Hoon, B.; Field, M.; Manouvrier-Hanu, S.; S, E. H.; Kammoun, M.; Gripp, K. W.; Bauer, C.; Schroeder, C.; Toutain, A.; Mihalic Mosher, T.; Kelly, B. J.; White, P.; Dufke, A.; Rentmeester, E.; Moon, S.; Koboldt, D. C.; van Roozendaal, K. E. P.; Hu, H.; Haas, S. A.; Ropers, H. H.; Murray, L.; Haan, E.; Shaw, M.; Carroll, R.; Friend, K.; Liebelt, J.; Hobson, L.; De Rademaeker, M.; Geraedts, J.; Fryns, J. P.; Vermeesch, J.; Raynaud, M.; Riess, O.; Gribnau, J.; Katsanis, N.; Devriendt, K.; Bauer, P.; Gecz, J.; Golzio, C.; Gontan, C.; Kalscheuer, V. M., Pathogenic variants in E3 ubiquitin ligase RLIM/RNF12 lead to a syndromic X-linked intellectual disability and behavior disorder. *Mol Psychiatry* **2019,** *24* (11), 1748-1768, doi:10.1038/s41380-018-0065-x.

186. Wang, F.; McCannell, K. N.; Boskovic, A.; Zhu, X.; Shin, J.; Yu, J.; Gallant, J.; Byron, M.; Lawrence, J. B.; Zhu, L. J.; Jones, S. N.; Rando, O. J.; Fazzio, T. G.; Bach, I., Rlim-Dependent and -Independent Pathways for X Chromosome Inactivation in Female ESCs. *Cell Rep* **2017,** *21* (13), 3691-3699, doi:10.1016/j.celrep.2017.12.004.

187. Chen, X.; Liu, L.; Zeng, C., A novel variant in SLC16A2 associated with typical Allan-Herndon-Dudley syndrome: a case report. *BMC Pediatr* **2022,** *22* (1), 180, doi:10.1186/s12887-022-03259-5.

188. Nino-Soto, M. I.; Nuber, U. A.; Basrur, P. K.; Ropers, H. H.; King, W. A., Differences in the pattern of X-linked gene expression between fetal bovine muscle and fibroblast cultures derived from the same muscle biopsies. *Cytogenet Genome Res* **2005,** *111* (1), 57-64, doi:10.1159/000085671.

189. Li, Z.; Li, Z.; Wang, L.; Long, C.; Zheng, Z.; Zhuang, X., ZCCHC13-mediated induction of human liver cancer is associated with the modulation of DNA methylation and the AKT/ERK signaling pathway. *J Transl Med* **2019,** *17* (1), 108, doi:10.1186/s12967-019-1852-0.

190. Swathi, D.; Ramya, L.; Archana, S. S.; Lavanya, M.; Krishnappa, B.; Binsila, B. K.; Selvaraju, S., X chromosome-linked genes in the mature sperm influence semen quality and fertility of breeding bulls. *Gene* **2022,** *839*, 146727, doi:10.1016/j.gene.2022.146727.

191. CHIC1 GeneCards. https://www.genecards.org/cgi-bin/carddisp.pl?gene=CHIC1 (accessed on 21 July 2024).

192. CDX4 GeneCards. https://www.genecards.org/cgi-bin/carddisp.pl?gene=CDX4 (accessed 2024).

193. Wang, Z.; Day, N.; Trifillis, P.; Kiledjian, M., An mRNA stability complex functions with poly(A)-binding protein to stabilize mRNA in vitro. *Mol Cell Biol* **1999,** *19* (7), 4552-60, doi:10.1128/MCB.19.7.4552.
194. Yang, S.; Li, X.; Liu, X.; Ding, X.; Xin, X.; Jin, C.; Zhang, S.; Li, G.; Guo, H., Parallel comparative proteomics and phosphoproteomics reveal that cattle myostatin regulates phosphorylation of key enzymes in glycogen metabolism and glycolysis pathway. *Oncotarget* **2018,** *9* (13), 11352-11370, doi:10.18632/oncotarget.24250.
195. Chen, K.; Zhang, X.; Wu, Y. D.; Wiest, O., Inhibition and mechanism of HDAC8 revisited. *J Am Chem Soc* **2014,** *136* (33), 11636-43, doi:10.1021/ja501548p.
196. Rajawat, D.; Panigrahi, M.; Nayak, S. S.; Bhushan, B.; Mishra, B. P.; Dutt, T., Dissecting the genomic regions of selection on the X chromosome in different cattle breeds. *3 Biotech* **2024,** *14* (2), 50, doi:10.1007/s13205-023-03905-4.
197. Lu, Z.; Fei, L.; Hou, G., A pan-cancer analysis of the oncogenic role of ERCC6L. *BMC Cancer* **2022,** *22* (1), 1347, doi:10.1186/s12885-022-10452-3.
198. PIN4 GeneCards. https://www.genecards.org/cgi-bin/carddisp.pl?gene=PIN4 (accessed on 21 July 2024).
199. Irie, M.; Itoh, J.; Matsuzawa, A.; Ikawa, M.; Kiyonari, H.; Kihara, M.; Suzuki, T.; Hiraoka, Y.; Ishino, F.; Kaneko-Ishino, T., Retrovirus-derived RTL5 and RTL6 genes are novel constituents of the innate immune system in the eutherian brain. *Development* **2022,** *149* (18), doi:10.1242/dev.200976.
200. Kaneko-Ishino, T.; Ishino, F., Retrovirus-Derived RTL/SIRH: Their Diverse Roles in the Current Eutherian Developmental System and Contribution to Eutherian Evolution. *Biomolecules* **2023,** *13* (10), doi:10.3390/biom13101436.
201. Kuo, P. T.; Zeng, Z.; Salim, N.; Mattarollo, S.; Wells, J. W.; Leggatt, G. R., The Role of CXCR3 and Its Chemokine Ligands in Skin Disease and Cancer. *Front Med (Lausanne)* **2018,** *5*, 271, doi:10.3389/fmed.2018.00271.
202. Kaushik, A. K.; Kandavel, H.; Nalpathamkalam, T.; Pasman, Y., Bovine neonate is deficient in innate immunity at birth. *Mol Immunol* **2021,** *133*, 101-109, doi:10.1016/j.molimm.2021.02.005.
203. Ribeiro, J.; Crossan, G. P., GCNA is a histone binding protein required for spermatogonial stem cell maintenance. *Nucleic Acids Res* **2023,** *51* (10), 4791-4813, doi:10.1093/nar/gkad168.
204. Zhang, N.; Jiang, H.; Zhang, K.; Zhu, J.; Wang, Z.; Long, Y.; He, Y.; Feng, F.; Liu, W.; Ye, F.; Qu, W., OGT as potential novel target: Structure, function and inhibitors. *Chem Biol Interact* **2022,** *357*, 109886, doi:10.1016/j.cbi.2022.109886.
205. Wang, T. F.; Feng, Z. Q.; Sun, Y. W.; Zhao, S. J.; Zou, H. Y.; Hao, H. S.; Du, W. H.; Zhao, X. M.; Zhu, H. B.; Pang, Y. W., Disruption of O-GlcNAcylation Homeostasis Induced Ovarian Granulosa Cell Injury in Bovine. *Int J Mol Sci* **2022,** *23* (14), doi:10.3390/ijms23147815.
206. Gudmundsson, S.; Wilbe, M.; Filipek-Gorniok, B.; Molin, A. M.; Ekvall, S.; Johansson, J.; Allalou, A.; Gylje, H.; Kalscheuer, V. M.; Ledin, J.; Anneren, G.; Bondeson, M. L., TAF1, associated with intellectual disability in humans, is essential for embryogenesis and regulates neurodevelopmental processes in zebrafish. *Sci Rep* **2019,** *9* (1), 10730, doi:10.1038/s41598-019-46632-8.
207. Zhang, J.; Huang, L.; Zhang, P.; Huang, X.; Yang, W.; Liu, R.; Sun, Q.; Lu, Y.; Zhang, M.; Fu, Q., Genomic Identification, Evolution, and Expression Analysis of Bromodomain Genes Family in Buffalo. *Genes (Basel)* **2022,** *13* (1), doi:10.3390/genes13010103.

208. ITGB1BP2 National Library of Medicine. (https://www.ncbi.nlm.nih.gov/gene/26548 (accessed on 21 July 2024).
209. Roessler, F.; Beck, A. E.; Susie, B.; Tobias, B.; Begtrup, A.; Biskup, S.; Caluseriu, O.; Delanty, N.; Frohlich, C.; Greally, M. T.; Karnstedt, M.; Klockner, C.; Kurtzberg, J.; Schubert, S.; Schulze, M.; Weidenbach, M.; Westphal, D. S.; White, M.; Wolf, C. M.; Zyskind, J.; Popp, B.; Strehlow, V., Genetic and phenotypic spectrum in the NONO-associated syndromic disorder. *Am J Med Genet A* **2023,** *191* (2), 469-478, doi:10.1002/ajmg.a.63044.
210. Afshar, H.; Khamse, S.; Alizadeh, F.; Delbari, A.; Najafipour, R.; Bozorgmehr, A.; Khazaei, M.; Adelirad, F.; Alizadeh, A.; Kowsari, A.; Ohadi, M., Evolving evidence on a link between the ZMYM3 exceptionally long GA-STR and human cognition. *Sci Rep* **2020,** *10* (1), 19454, doi:10.1038/s41598-020-76461-z.
211. Hu, X.; Shen, B.; Liao, S.; Ning, Y.; Ma, L.; Chen, J.; Lin, X.; Zhang, D.; Li, Z.; Zheng, C.; Feng, Y.; Huang, X.; Han, C., Gene knockout of Zmym3 in mice arrests spermatogenesis at meiotic metaphase with defects in spindle assembly checkpoint. *Cell Death Dis* **2017,** *8* (6), e2910, doi:10.1038/cddis.2017.228.
212. GJB1 National Library of Medicine. https://www.ncbi.nlm.nih.gov/gene/2705 (accessed on 21 July 2024).
213. Ibeagha-Awemu, E. M.; Bissonnette, N.; Bhattarai, S.; Wang, M.; Dudemaine, P. L.; McKay, S.; Zhao, X., Whole Genome Methylation Analysis Reveals Role of DNA Methylation in Cow's Ileal and Ileal Lymph Node Responses to Mycobacterium avium subsp. paratuberculosis Infection. *Front Genet* **2021,** *12*, 797490, doi:10.3389/fgene.2021.797490.
214. Ahmed, N.; Iu, J.; Brown, C. E.; Taylor, D. W.; Kandel, R. A., Serum- and growth-factor-free three-dimensional culture system supports cartilage tissue formation by promoting collagen synthesis via Sox9-Col2a1 interaction. *Tissue Eng Part A* **2014,** *20* (15-16), 2224-33, doi:10.1089/ten.TEA.2013.0559.
