# Supplementary material for: Simultaneous Effects of Single-Nucleotide Polymorphisms on the Estimated Breeding Value of Milk, Fat, and Protein Yield of Holstein Friesian Cows in Hungary
Source: Animals (Basel). 2024 Dec 5;14(23):3518. doi: 10.3390/ani14233518 (PMC11640446; doi:10.3390/ani14233518)
Supplement: Supplementary file 1 [file animals-14-03518-s001.zip › Table S1.pdf]

Table S1. The names of the markers associated with two or three EBV values, their genomic positions (*B. taurus* genome build ARS-UCD1.2), and genes found around the markers. Single SNPs or groups of SNPs located close to each other are separated by horizontal lines. Underlined genes play roles in calcium transport, starred ones are in collagen related processes. The cfm label is for the genes with known cilia and flagella functions and/or known as membrane proteins.

Marker name	Chr	Position (bp)	EBV			± 1 million bp	± 3 million bp
			milk	fat	prot		
BTA-38502-no-rs	1	82,339,579	+		+	*C1H3orf70*	
ARS-BFGL-NGS-14913	1	82,360,713	+		+	*LINC02054, CLCN2, MAP6D1, YEATS2*	
ARS-BFGL-NGS-110543	1	86,233,203	+		+	*TTC14*[cfm]	
BovineHD0100037693	1	131,261,458	+		+	*ARMC8, CEP70*	
BovineHD0100037725	1	131,389,856	+		+		
BovineHD0100037732	1	131,413,283	+		+		
BovineHD0100037734	1	131,427,641	+		+		
ARS-BFGL-NGS-37290	1	136,049,399	+		+	*TOPBP1*	
BTB-00039698	1	136,090,182	+		+		
ARS-BFGL-NGS-78397	1	137,305,479	+		+		
Hapmap51079-BTA-88097	2	20,011,118	+		+	*HOXD3*	
Hapmap47966-BTA-47563	2	48,824,025	+	+	+		
ARS-BFGL-NGS-113042	2	48,993,143	+	+	+		
BTB-01405574	3	40,190,639	+		+	*RNPC3*	
BTB-01982674	3	61,970,189		+	+		
BTB-00134966	3	69,934,639		+	+		
BTB-00135076	3	69,961,802		+	+		
BTB-01393342	3	71,022,745		+	+		
Hapmap57979-rs29017982	3	73,879,769		+	+		
Hapmap43144-BTA-107773	3	73,904,881		+	+	*CTH*	
BTB-00182731	4	46,190,578	+		+		*PHTF2*
BTB-00182813	4	46,311,240	+		+	*EFCAB10*	
ARS-BFGL-NGS-30059	4	46,943,050	+		+		
BTB-01637746	4	47,751,036	+		+		
BTB-00219372	5	9,085,964	+	+	+		*PPFIA2, METTL25*
EuroG10K_chr5_106240327	5	105,749,785	+		+		*TAPBPL**
EuroGMD_DEN_QGG_5_106252827	5	105,762,284	+		+		
DBR_Chr5_106260278_rs109351328	5	105,769,735	+		+		

EuroGMD_DEN_QGG_5_106260278	5	105,769,735	+		+		
EuroGMD_DEN_QGG_5_106261873	5	105,771,330	+		+		
EuroGMD_DEN_QGG_5_106262740	5	105,772,197	+		+		
EuroGMD_DEN_QGG_5_106263967	5	105,773,382	+		+		
BovineHD0500030487	5	105,773,809	+		+		
EuroGMD_DEN_QGG_5_106264394	5	105,773,809	+		+		
EuroG10K_chr5_106267060	5	105,776,475	+		+		
DB-364-seq-rs378727865	5	105,784,987	+		+	*TSPAN11*	*FBXL14*
Hapmap47766-BTA-87827	6	100,139,940	+		+		*HNRNPD, HNRNPDL, KLHL8*
EuroGMD_DEN_QGG_9_38739113	9	38,271,438	+	+		*TUBE1, MFSD4B*	
EuroG10K_BTA-04956-no-rs	11	94,715,801	+		+	*STRBP*	
ARS-BFGL-NGS-98451	11	97,225,356	+		+		*STRBP*
ARS-BFGL-NGS-83830	11	102,752,125		+	+	*NTNG2, TTF1, SPACA9*[cfm]*, PAEP, KCNT1, CARD9*	
Hapmap36617-SCAFFOLD188701_463	13	34,319,303	+		+		*PTCHD3*
ARS-BFGL-NGS-52422	14	37,166,868	+		+	*SBSPON, TMEM70, JPH1*	
SNP_1KG_14_37273185	14	37,273,185	+		+		
Hapmap34185-BES7_Contig323_940	15	55,356,241	+		+		*ARAP1, P2RY2, P4HA3*, MYO7A*
ARS-BFGL-NGS-118490	18	12,718,603	+		+	*EMC8*	
ARS-BFGL-NGS-64457	18	12,739,390	+		+		
BTB-01627667	18	21,596,766	+	+			*NOD2*
ARS-BFGL-BAC-36240	18	21,636,399	+	+			
Hapmap42547-BTA-42724	18	21,791,294	+	+			
Hapmap35910-SCAFFOLD37470_667	19	31,569,771	+		+		
UA-IFASA-7101	19	32,389,986	+		+		
ARS-BFGL-NGS-100358	19	32,754,596	+	+			
ARS-BFGL-NGS-116379	19	32,997,278	+		+		
ARS-BFGL-NGS-110037	19	33,052,413	+		+	*ZNF624, CENPV, ULK2*	
Hapmap32042-BTA-133010	19	33,716,989		+	+	*ALDH3A2, B9D1*[cfm]*, PRPSAP2, MYO15A, TOM1L2*	
ARS-BFGL-NGS-34178	22	10,597,656		+	+	*C22H3orf35, DLEC1*[cfm]	
ARS-BFGL-NGS-21216	22	11,596,830		+	+	*MYD88*	
ARS-BFGL-NGS-65384	22	11,862,971		+	+	*SLC22A13, ACVR2B, EXOG*	
ARS-BFGL-NGS-104806	22	12,291,232		+	+	*MOBP*	
ARS-BFGL-NGS-15552	22	14,961,300		+	+	*CTNNB1, CCDC13*[cfm]*, ACKR2*	
ARS-BFGL-NGS-4910	22	16,130,993		+	+	*ZNF852, TATDN2, RPUSD3, TTLL3, SETD5*	
ARS-BFGL-NGS-24520	22	18,761,349		+	+	*SRGAP3, OXTR*	
ARS-BFGL-BAC-28665	24	28,487,771	+		+		*ZNF503*
EuroG10K_ARS-BFGL-NGS-109112	28	36,015,224	+	+	+		
ARS-BFGL-NGS-33494	28	36,097,359	+	+	+		
Hapmap51965-BTA-101198	28	36,110,502	+		+		
ARS-BFGL-NGS-83238	28	36,205,983	+		+		

BTA-64158-no-rs	28	37,195,142	+	+	+		*CCSER2, SHLD2, ANXA8L1*
Hapmap46921-BTA-106251	X	30,978,737	+	+	+	*FMR1, FMR1NB, AFF2, IDS, CXHXorf40A, TMEM185A*	
Hapmap60788-rs29017234	X	77,312,570		+	+	*NEXMIF, RLIM, SLC16A2, ZCCHC13, CHIC1, CDX4, MGC140080, PABPC1L2A, PHKA1, HDAC8*	*CITED1, RPS4X, ERCC6L, PIN4, NHSL2, RTL5, CXCR3, GCNA, OGT, TAF1, ITGB1BP2, NONO, ZMYM3, GJB1, NLGN3, MED12*
Hapmap49448-BTA-111996	X	87,848,657	+		+	*GLOD5, GATA1, HDAC6, ERAS, PCSK1N, TIMM17B, PQBP1, SLC35A2, PIM2, OTUD5, KCND1, GRIPAP1, TFE3, CCDC120, PRAF2, WDR45, GPKOW, PLP2, PRICKLE3, SYP, CACNA1F, CCDC22, FOXP3, PPP1R3F, PAGE4, USP27X, CLCN5*	*SLC38A5, FTSJ1, PORCN, EBP, TBC1D25, RBM3, WDR13, WAS, SUV39H1*
ARS-BFGL-NGS-10300	X	87,915,822	+	+	+	*AKAP4, CCNB3, DGKK, SHROOM4, BMP15, NUDT10*	*CXHXorf67, NUDT11, GSPT2, MAGED1, MAGED4B*
BovineHD3000027615	X	95,636,192	+	+	+	*HEPH, VSIG4, MSN, LAS1L, ZC3H12B, ZC4H2, ASB12, AMER1, ARHGEF9*	*SPIN4, ZXDB, ZNF674*
