# Supplementary material for: Simultaneous Effects of Single-Nucleotide Polymorphisms on the Estimated Breeding Value of Milk, Fat, and Protein Yield of Holstein Friesian Cows in Hungary
Source: Animals (Basel). 2024 Dec 5;14(23):3518. doi: 10.3390/ani14233518 (PMC11640446; doi:10.3390/ani14233518)
Supplement: Supplementary file 1 [file animals-14-03518-s001.zip › Table S2.pdf]

Table S2. The means of rescaled values of the applied algorithms and regression beta values of linear regressions on EBVs for MY, FY, and PY. Means >0.8 are shown in bold. The regression beta values for SNPs whose effect direction differed between MY, FY, and PY are also shown in bold.

				means			Reg.Beta	
**Marker name**	**Chr**	**Position (bp)**	**milk**	**fat**	**prot**	**milk**	**fat**	**prot**
BTA-38502-no-rs	1	82,339,579	0.7668		**0.8850**	0.2926		0.3150
ARS-BFGL-NGS-14913	1	82,360,713	0.7427		**0.8804**	0.2724		0.3006
ARS-BFGL-NGS-110543	1	86,233,203	0.5700		0.4984	-0.2157		-0.2167
BovineHD0100037693	1	131,261,458	0.6135		0.4454	0.2750		0.2249
BovineHD0100037725	1	131,389,856	0.5922		0.6775	-0.2426		-0.3005
BovineHD0100037732	1	131,413,283	0.6472		0.7173	-0.2548		-0.3103
BovineHD0100037734	1	131,427,641	0.6299		0.7224	-0.2478		-0.3087
ARS-BFGL-NGS-37290	1	136,049,399	0.6173		0.5302	0.2585		0.2305
BTB-00039698	1	136,090,182	0.6173		0.5333	0.2585		0.2316
ARS-BFGL-NGS-78397	1	137,305,479	0.6890		0.6020	0.3298		0.2981
Hapmap51079-BTA-88097	2	20,011,118	0.5978		0.5888	-0.2179		-0.2352
Hapmap47966-BTA-47563	2	48,824,025	0.7321	0.6739	0.5653	-0.2508	-0.2331	-0.2368
ARS-BFGL-NGS-113042	2	48,993,143	**0.8243**	0.7631	0.5296	0.2656	0.2505	0.2254
BTB-01405574	3	40,190,639	0.6453		0.5934	-0.2412		-0.2611
BTB-01982674	3	61,970,189		0.6965	0.5421		0.2209	0.1972
BTB-00134966	3	69,934,639		0.6459	0.4890		0.2328	0.2184
BTB-00135076	3	69,961,802		0.6733	0.4820		0.2381	0.2197
BTB-01393342	3	71,022,745		0.5919	0.5536		0.2557	0.2578
Hapmap57979-rs29017982	3	73,879,769		**0.9021**	0.7212		-0.2956	-0.2887
Hapmap43144-BTA-107773	3	73,904,881		**0.9100**	0.7127		-0.2940	-0.2843
BTB-00182731	4	46,190,578	0.6399		0.5215	0.2512		0.2499
BTB-00182813	4	46,311,240	0.6291		0.5190	0.2499		0.2499
ARS-BFGL-NGS-30059	4	46,943,050	0.7054		0.5500	0.2577		0.2545
BTB-01637746	4	47,751,036	0.7527		0.5175	0.2384		0.2278
BTB-00219372	5	9,085,964	0.6956	0.7898	0.5834	**0.2332**	**-0.2725**	**-0.2362**
EuroG10K_chr5_106240327	5	105,749,785	0.5999		0.5597	-0.4027		-0.3961
EuroGMD_DEN_QGG_5_106252827	5	105,762,284	0.5701		0.5477	-0.3634		-0.3856
DBR_Chr5_106260278_rs109351328	5	105,769,735	0.6168		0.5725	-0.4062		-0.4034
EuroGMD_DEN_QGG_5_106260278	5	105,769,735	0.6168		0.5725	-0.4062		-0.4034
EuroGMD_DEN_QGG_5_106261873	5	105,771,330	0.5701		0.5477	-0.3634		-0.3856
EuroGMD_DEN_QGG_5_106262740	5	105,772,197	0.5701		0.5477	-0.3634		-0.3856
EuroGMD_DEN_QGG_5_106263967	5	105,773,382	0.5701		0.5481	-0.3634		-0.3856
BovineHD0500030487	5	105,773,809	0.5701		0.5481	-0.3634		-0.3856
EuroGMD_DEN_QGG_5_106264394	5	105,773,809	0.5701		0.5481	-0.3634		-0.3856
EuroG10K_chr5_106267060	5	105,776,475	0.6168		0.5729	-0.4062		-0.4034
DB-364-seq-rs378727865	5	105,784,987	0.5658		0.5461	-0.3618		-0.3849

Hapmap47766-BTA-87827	6	100,139,940	0.7370		0.4623	-0.2490		-0.2081
EuroGMD_DEN_QGG_9_38739113	9	38,271,438	0.7574	0.6010		0.3173	0.2971	
EuroG10K_BTA-04956-no-rs	11	94,715,801	**0.8646**		0.5003	0.2578		0.2107
ARS-BFGL-NGS-98451	11	97,225,356	**0.8027**		0.4776	0.2630		0.2053
ARS-BFGL-NGS-83830	11	102,752,125		0.6571	0.6506		0.2543	0.2778
Hapmap36617-SCAFFOLD188701_463	13	34,319,303	0.7220		0.4398	0.2803		0.2454
ARS-BFGL-NGS-52422	14	37,166,868	0.6053		0.5841	0.2241		0.2257
SNP_1KG_14_37273185	14	37,273,185	0.5976		0.5708	0.2220		0.2254
Hapmap34185-BES7_Contig323_940	15	55,356,241	0.6617		0.4753	-0.3369		-0.3017
ARS-BFGL-NGS-118490	18	12,718,603	0.7949		0.5844	-0.2317		-0.2198
ARS-BFGL-NGS-64457	18	12,739,390	0.7497		0.5761	-0.2215		-0.2169
BTB-01627667	18	21,596,766	0.7656	0.5947		-0.2287	-0.2232	
ARS-BFGL-BAC-36240	18	21,636,399	0.7722	0.5953		-0.2287	-0.2232	
Hapmap42547-BTA-42724	18	21,791,294	0.7931	0.7306		0.2329	0.2502	
Hapmap35910-SCAFFOLD37470_667	19	31,569,771	**0.9325**		0.4704	-0.2736		-0.2033
UA-IFASA-7101	19	32,389,986	0.5894		0.5096	-0.2218		-0.2185
ARS-BFGL-NGS-100358	19	32,754,596	0.7236	0.7733		-0.2312	-0.2633	
ARS-BFGL-NGS-116379	19	32,997,278	0.6596		0.5390	-0.2281		-0.2378
ARS-BFGL-NGS-110037	19	33,052,413	0.6940		0.6037	-0.2337		-0.2501
Hapmap32042-BTA-133010	19	33,716,989		0.6163	0.4525		-0.3042	-0.2666
ARS-BFGL-NGS-34178	22	10,597,656		0.7130	0.5641		-0.2602	-0.2403
ARS-BFGL-NGS-21216	22	11,596,830		0.6881	0.5993		0.2580	0.2556
ARS-BFGL-NGS-65384	22	11,862,971		0.7445	0.6077		0.2656	-0.2572
ARS-BFGL-NGS-104806	22	12,291,232		0.6404	0.5233		-0.2363	-0.2280
ARS-BFGL-NGS-15552	22	14,961,300		0.6521	0.4941		0.2798	0.2369
ARS-BFGL-NGS-4910	22	16,130,993		0.6651	0.5185		0.2643	0.2294
ARS-BFGL-NGS-24520	22	18,761,349		**0.8991**	0.4542		0.3031	0.2328
ARS-BFGL-BAC-28665	24	28,487,771	0.6145		0.4514	0.2651		0.2479
EuroG10K_ARS-BFGL-NGS-109112	28	36,015,224	**0.8100**	0.5828	**0.9359**	-0.2848	-0.2371	-0.3302
ARS-BFGL-NGS-33494	28	36,097,359	**0.8524**	0.6801	**0.9310**	-0.2561	-0.2410	-0.2988
Hapmap51965-BTA-101198	28	36,110,502	**0.8563**		**0.8292**	-0.2521		-0.2616
ARS-BFGL-NGS-83238	28	36,205,983	0.6997		0.6887	-0.2498		-0.2493
BTA-64158-no-rs	28	37,195,142	**0.9260**	**0.8600**	**0.8761**	-0.2722	-0.2762	-0.2726
Hapmap46921-BTA-106251	X	30,978,737	0.6630	**0.9791**	0.5772	0.2263	0.2857	0.2338
Hapmap60788-rs29017234	X	77,312,570		**0.9058**	0.4445		0.3222	0.2439
Hapmap49448-BTA-111996	X	87,848,657	0.7747		0.4882	-0.2287		-0.2012
ARS-BFGL-NGS-10300	X	87,915,822	**0.9348**	**0.9237**	0.5436	0.2584	0.2952	0.2282
BovineHD3000027615	X	95,636,192	**0.8607**	0.6759	0.4581	**0.2432**	**-0.2187**	**0.1896**
